# Systematic review and meta-analysis of cardiovascular associated biomarkers in adults with asymptomatic autoimmune diseases

**DOI:** 10.3389/fcvm.2025.1598590

**Published:** 2025-10-16

**Authors:** Waiseng U, Lin Hong, Harshawardhan Dhanraj Ramteke, Jiaen Yang, Zhiwei He

**Affiliations:** ^1^ The First Clinical Medical College, Guangdong Medical University, Zhanjiang, Guangdong, China; ^2^ Department of Pharmacy, Ezhou Central Hospital, Ezhou, Hubei, China; ^3^ School of International Education, Anhui Medical University, Hefei, Anhui, China; ^4^ Department of Rehabilitation, The People's Hospital of Gaoming District of Foshan City, Foshan, Guangdong, China; ^5^ School of Basic Medicine Science, Guangdong Medical University, Dongguan, Guangdong, China

**Keywords:** autoimmune disease, cardiovascular disease, advanced biomarkers, prediction, atherosclerosis

## Abstract

**Introduction:**

Cardiovascular disease (CVD) remains a leading cause of death. Autoimmune patients face heightened CVD risk due to chronic inflammation and immune dysregulation. This systematic review and meta-analysis aim to synthesize current evidence on the predictive value of advanced or novel autoimmune biomarkers for the occurrence of CVD in middle-aged patients with autoimmune diseases and without cardiovascular history or symptoms.

**Methods:**

Abstract was registered prospectively (PROSPERO CRD42024611894) and conducted an advanced, MeSH-based search (2004–2025) for studies on autoimmune diseases in adults (18–65) without prior CVD, in various databases. Pooled adjusted hazard ratios were generated using Stata 18, assessing heterogeneity (Cochran's *Q*, *I*^2^), publication bias (funnel plot, Egger's test), and risk of bias (ROBINS-I), with sensitivity analysis performed to confirm robustness.

**Result:**

A comprehensive search in PubMed, Embase, and Medline yielded 3,975 records (after removing 237 duplicates), and after screening 2,488 titles/abstracts and 896 full texts, 69 studies (34 for meta-analysis) with 46,493 participants were included after excluding 188 with pre-existing CVD and 117 with insufficient data. High-sensitivity C-reactive protein (hs-CRP) was consistently associated with elevated CVD risk despite high heterogeneity and potential publication bias. Similarly, lupus anticoagulant, sVCAM-1, and antiphospholipid antibodies demonstrated strong predictive associations. In contrast, rheumatoid factor, anti-CCP, ADMA, homocysteine, NT-proBNP, anti-dsDNA, and TNF-alpha showed borderline significance or inconsistent results. These findings underscore the potential of select inflammatory and immune markers for enhancing CVD risk stratification and guiding targeted prevention strategies.

**Conclusion:**

Integrating these biomarkers with traditional risk factors may enable early detection of subclinical atherosclerosis in autoimmune patients, pending further research.

**Systematic Review Registration:**

https://www.crd.york.ac.uk/PROSPERO/view/CRD42024611894, identifier CRD42024611894.

## Graphical Abstract

**Figure:**
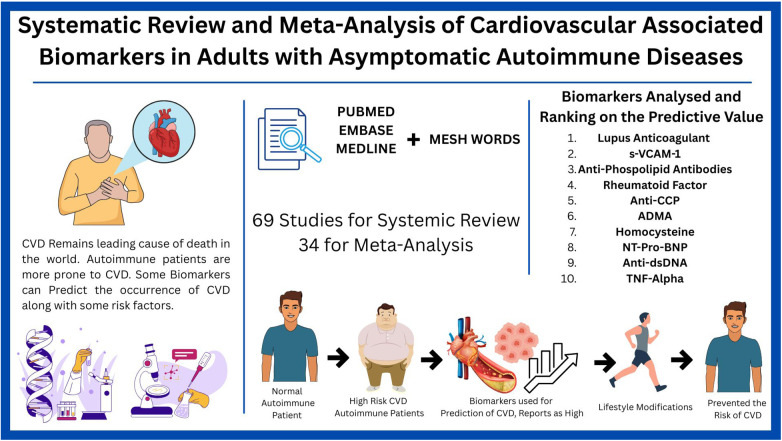
Created using Canva, licensed under Free Content License.

## 1 Introduction

Introduction needs some additional information about the studied biomarkers throughout the manuscript, what are they biologically, when have they increased and how to be used for differential diagnosis as well.

Healthcare professionals, researchers, and public health organizations have invested significant efforts in mitigating the impact of cardiovascular disease (CVD); however, it remains one of the leading causes of illness and death worldwide (1). In adults with autoimmune disorders, conventional risk factors such as dyslipidemia, hypertension, and hyperglycemia may interact synergistically with the chronic inflammatory state and immune dysregulation inherent in these conditions, potentially increasing the risk of cardiovascular disease (2). Given the significant cardiovascular burden in autoimmune patients, extensive research now focuses on novel biomarkers and imaging techniques that may offer more accurate outcome prediction and earlier disease detection of CVD. This strategy empowers even those without obvious risk factors to assess their risk early and adopt healthier lifestyle habits which later prevents the risk by 30% (3).

Atherosclerotic cardiovascular disease significantly burdens the general population, but chronic inflammation in autoimmune patients further elevates their risk, underscoring the need for early detection and tailored management (4). In autoimmune conditions like Systemic Lupus Erythematous (SLE), Rheumatoid Arthritis (RA), or vasculitis, atherosclerosis is fueled by lipid buildup, while immune dysregulation intensifies inflammatory responses, oxidative stress, and endothelial dysfunction, accelerating damage to vascular endothelial lining (5). Thus, autoimmune markers [Rheumatoid Factor (RF), high-sensitivity C-reactive protein (hsCRP), lupus anticoagulant (LA), homocysteine (Hcy), anti-cyclic citrullinated peptide (anti-CCP), interleukin-6 (IL-6), asymmetrical dimethylarginine (ADMA), anti-double stranded DNA (anti-dsDNA), soluble vascular adhesion molecule-1 (sVCAM-1), N-terminal pro b-type natriuretic peptide (NT-proBNP), antiphospholipid antibodies (APL), tumor necrosis factor-alpha (TNF-α), fibrinogen, and anticardiolipin antibodies (aCL)] have been proposed as additional tools to predict CVD in autoimmune patients (6–10). In brief, RF is an autoantibody that binds to IgG, triggering inflammation and increasing CVD risk in autoimmune conditions. hsCRP reflects systemic inflammation and helps assess CVD risk, particularly in autoimmune patients. LA and aPL increase thrombotic risk, aiding in the diagnosis of clotting events. Elevated Hcy levels disrupt endothelial function and promote atherosclerosis, assisting in early CVD detection in autoimmune disorders. Anti-CCP and anti-dsDNA antibodies are markers for active autoimmune diseases like RA and lupus, linking inflammation to endothelial damage. IL-6 exacerbates endothelial injury, promoting atherosclerosis in autoimmune conditions. ADMA signals early vascular damage by inhibiting nitric oxide production. sVCAM-1 indicates endothelial activation, aiding in early CVD detection. Elevated NT-proBNP levels reflect myocardial stress in autoimmune patients. TNF-α and Fibrinogen are inflammatory markers that contribute to atherosclerosis and thrombotic risks, guiding treatment strategies in autoimmune CVD. Together, these biomarkers are essential for differential diagnosis, helping distinguish cardiovascular events related to autoimmune inflammation from those caused by traditional atherosclerotic processes. Similarly, the ability of autoimmune markers to predict cardiovascular events has been validated using widely recognized predictors, such as high-sensitive troponin (hsTn), NT-proBNP (11, 12).

This approach underscores that autoimmune markers can be as effective as conventional indicators in refining cardiovascular risk assessment. Numerous studies have explored the potential of various biomarkers to forecast cardiovascular disease and mortality in high-risk autoimmune groups; however, most research has focused on those already recognized as at increased risk. Understanding the predictive value of autoimmune biomarkers requires considering the intricate interactions among chronic systemic inflammation, immune dysregulation, aging, and underlying disease activity. There is limited guidance on whether these biomarkers can reliably predict CVD and mortality in asymptomatic, middle-aged patients with autoimmune conditions. Likewise, a systematic review and meta-analysis in rheumatoid arthritis patients demonstrated that A systematic review and meta-analysis in rheumatoid arthritis (RA) patients demonstrated that biomarkers such as rheumatoid factor (RF), when compared to cardiac biomarkers like high-sensitivity troponin (hsTn) and B-type natriuretic peptide (BNP), effectively predicted subsequent cardiovascular events by reflecting underlying inflammation and myocardial injury. However, the study's focus was limited to RA populations (13). These findings suggest that such biomarkers could significantly improve early detection of elevated cardiovascular risk in autoimmune patients—risk that conventional screening methods might overlook. Therefore, this systematic review and meta-analysis aims to synthesize current evidence on the role of advanced and novel autoimmune biomarkers in predicting incident cardiovascular disease (CVD) among middle-aged autoimmune patients without prior cardiovascular history or symptoms.

## 2 Methods

**Figure 1:**
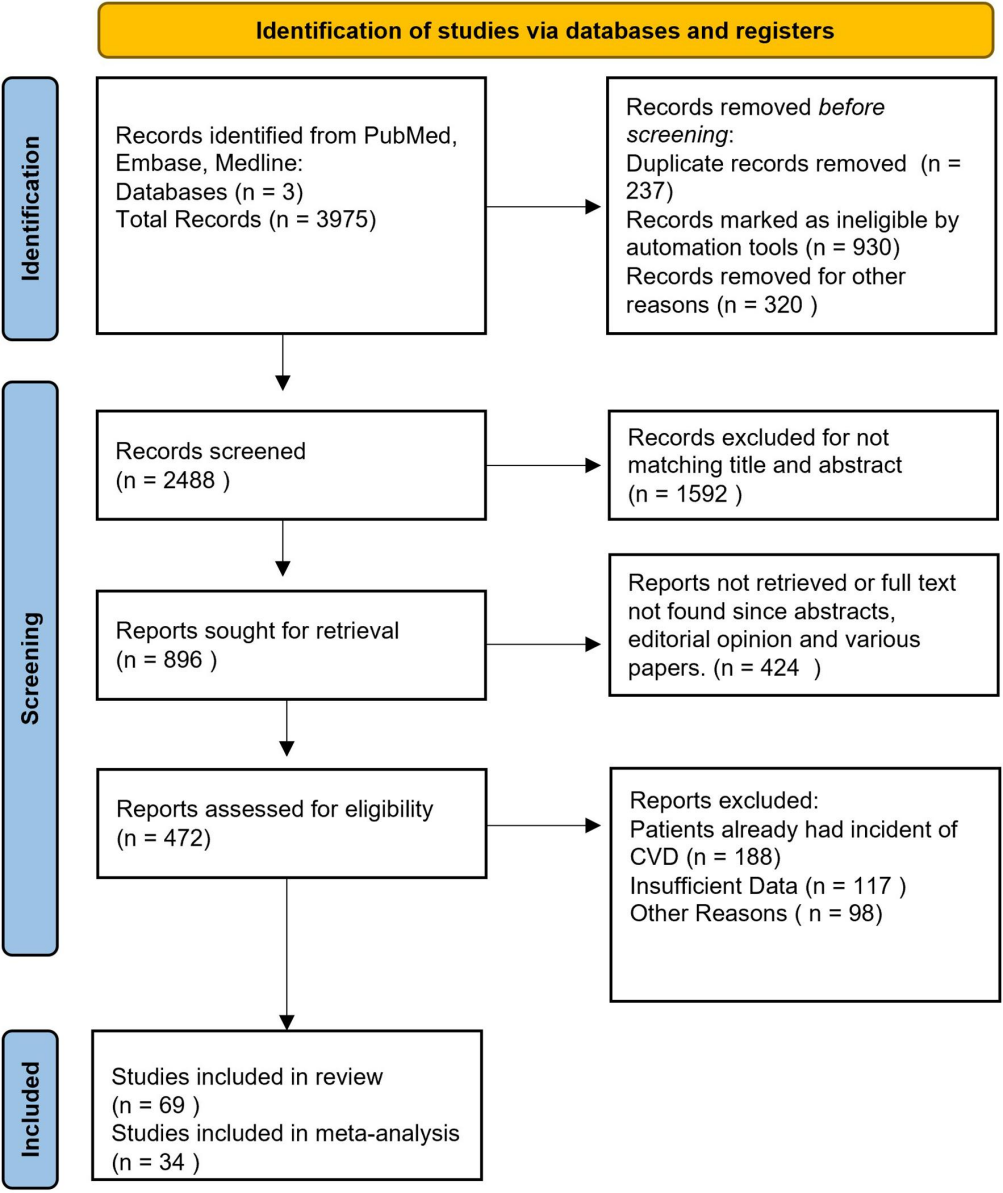
Prisma flow diagram.

The review protocol was prospectively registered with PROSPERO (14) (identifier: CRD42024611894). A comprehensive MeSH-based search across multiple databases, including PubMed, Embase, and Medline, was conducted to identify studies investigating biomarkers—such as RF, hsCRP, lupus anticoagulant, homocysteine, anti-CCP, IL-6, ADMA, anti-dsDNA, sVCAM-1, NT-proBNP, APL, TNF-α, fibrinogen, and ACL—in relation to autoimmune diseases like rheumatoid arthritis, systemic lupus erythematosus, and vasculitis, and their association with cardiovascular outcomes, including coronary heart disease, heart failure, stroke, peripheral artery disease, and CVD mortality. Data collection was performed from 01 January 2004 to 31 January 2025, following the Preferred Reporting Items for Systematic Reviews and Meta-Analyses (PRISMA) reporting guidelines (15). Detailed in the Supplementary Material, the search parameters were restricted by language (English), publication date (01 January 2004 to 31 January 2025 Last Search was done on 20 March 2025), and participant age (18–65 years), and encompassed cross-sectional, case-control, and cohort studies. Only studies involving patients diagnosed with an autoimmune disease and with no prior history of cardiovascular disease were considered. A flow diagram summarizing the study selection process is provided in Figure 1.

### 2.2 Data extraction and quality assessment

Following the initial literature search, duplicate records, conference abstracts, editorials, and letters to the editor were excluded. Two independent investigators then screened article titles and abstracts to assess eligibility for further review, with inter-rater agreement evaluated and any discrepancies resolved by a senior investigator. The inclusion criteria encompassed studies involving individuals aged 18–65 diagnosed with an autoimmune disease, with no prior or current history of cardiovascular disease, focusing on case-control and cohort study designs that examined emerging, novel, or advanced biomarkers beyond traditional glucose and lipid profiles. Conversely, studies were excluded if participants were older than 65, had any history of cardiovascular disease, or if the study design comprised clinical trials, case reports, or meta-analyses. After full-text and Supplementary Material review, 3,906 articles were removed for not meeting the inclusion criteria. Data extraction captured publication details, participant characteristics and biomarker descriptions, cardiovascular outcomes, and the authors' conclusions. Study validity was assessed using the Grading of Recommendations, Assessment, Development, and Evaluations (GRADE) criteria (16).

### 2.3 Data synthesis and analysis

In our meta-analysis, we used the meta command in Stata 18.0 (Stata Corp LP, College Station, TX, USA) to generate pooled summary estimates for the risk of incident cardiovascular disease (CVD) for each biomarker, provided that at least two studies offered eligible data for middle-aged adults without a history or symptoms of CVD. Incident CVD was defined to include events such as myocardial infarction, coronary revascularization procedures, stroke, heart failure, cardiac arrest, peripheral artery disease, or CVD-related death. We combined adjusted hazard ratios (HRs) from studies that employed similar methodologies. To evaluate the variability between study findings, we employed both Cochran's *Q* test and the *I*^2^ statistic, with *I*^2^ thresholds of 25%, 50%, and 75% indicating low, moderate, and high heterogeneity, respectively. Based on the level of heterogeneity detected, we selected either a fixed-effects or a random-effects model, reporting the results with 95% confidence intervals. Publication bias was investigated through the use of a funnel plot synthesis along with Egger's regression test to statistically assess any asymmetry in the funnel plot. The risk of bias was further examined using the Risk of Bias in Non-Randomized Studies of Interventions (ROBINS-I) tool. Finally, sensitivity analysis was performed by re-running the meta-analysis after excluding the study with the greatest weight to determine whether this removal led to a reduction in heterogeneity.

## 3 Results

### 3.1 Systemic review

A comprehensive search of PubMed, Embase, and Medline yielded 3,975 records, with 237 duplicates removed prior to screening. Of the remaining 2,488 titles/abstracts screened, 896 full-text articles were retrieved and 472 were ultimately assessed for eligibility. After excluding studies with patients who already had CVD (*n* = 188) and those with insufficient data (*n* = 117), 69 studies were included in the review, with 34 contributing to the meta-analysis. Ultimately, studies met all inclusion criteria with a total sample size of 46,493 participants. These included 38 were Case Control Studies, 28 Cohort Studies, 3 case-control studies (17–84). Most of the studies were rated Moderate in GRADE analysis and the quality of the studies were Good. The Biomarkers most commonly reported in these studies were hsCRP (*n* = 24), RF (*n* = 6), IL-6 (*n* = 6), Anti-CCP (*n* = 6), ADMA (*n* = 6), Hcy (*n* = 5), sVCAM-1 (*n* = 5), NT-proBNP (*n* = 4), APL (*n* = 4), Fibrinogen (*n* = 4), TNF-alpha (*n* = 3) and ACL (*n* = 2). Type of Study, Duration of Study, GRADE, Quality of Study, Biomarker, whether or not CVD Predicted, HTN, T2DM, Total Sample Population and what were the Main Findings are tabulated in Table 1.

**Table 1:** Summary of all papers included.

References	Autor Name/Year	Type of study	Duration of study	GRADE	Quality of study	Biomarker	CVD predicted	HTN	T2DM	Total sample	Main findings
Aiewruengsurat et al. (17)	Aiewruengsurat et al. 2023	Cross-sectional study	0.7 years	Moderate	Good	RF, ACL Antibody, High Sensitive Trop I, NT-proBNP	Yes	20	7	299	Weak correlations were found between cardiac biomarkers (hsTropT and NT-proBNP) and cardiac anatomy/function in RA patients without overt cardiovascular disease. hsTropT showed a weak positive correlation with LVMI and E/e’. Positive ACPA and higher hsTropT may increase LVMI. Factors such as systolic blood pressure and NSAID use contributed to increased LVMI. Aging was associated with higher E/e’ values.
Ajeganova et al. (18)	Ajeganova et al. 2013	Cohort Study	10 years	Moderate	Good	RF, ACL Antibody, hsCRP	Yes	224	53	741	RF or ACPA positivity and high inflammatory markers (CRP, ESR) were associated with higher CVD risk and mortality in patients with RA onset before age 65. Early reduction in inflammatory markers and disease activity improved long-term outcomes, particularly in older patients.
Arnab et al. (19)	Arnab et al. 2013	Cross-sectional study	1 Year	Moderate	Good	Anti-CCP, hsCRP	Yes	N/A	N/A	80	Anti-CCP antibodies are associated with Increased carotid intima-medial thickness (atherosclerosis marker), Higher CRP and ESR levels. Lower left ventricular ejection fraction.Greater prevalence of diastolic dysfunction and mild pericardial thickening. Anti-CCP-positive patients had worse cardiac and vascular profiles compared to anti-CCP-negative patients.
Bakry et al. (20)	Bakry et al. 2017	Cross-sectional study	2.5 Years	Low	Good	Interleukin (IL)-1β, IL-6, IL-18, C-reactive protein (CRP)	Yes	N/A	N/A	80	RA patients had significantly higher levels of IL-1β, IL-6, and IL-18 compared to controls. Disease activity (DAS-28) and RF positivity were major risk factors for ischemic heart disease in RA. Better disease control and remission can reduce cardiac complications in RA patients.
Bana et al. (21)	Bana et al. 2023	Cross-sectional study	3-8 years	Low	Good	IL-1B, IL-6, TNF-α, IFN-*γ*, TIMP-1, galectin-3, VCAM-1	Yes	N/A	N/A	49	Young SLE patients with subclinical cardiovascular disease (CVD) showed evidence of ongoing immune activation. Low MMP-9 levels may serve as early markers of myocardial disease. Cytokines (e.g., IL-1B, IL-6) and fibrosis markers (e.g., TIMP-1) were significantly elevated in SLE patients compared to controls.
Barbarroja et al. (22)	Barbarroja et al. 2014	Cohort Study	2 years	Low	Good	Anti-ccp, CRP	Yes	21	7	75	High titers of anti-CCPs are linked with altered expression of prothrombotic, inflammatory, and oxidative stress markers. Anti-CCPs promote leukocyte activation and contribute to the inflammatory and proatherogenic profile in rheumatoid arthritis (RA) patients​
Bengtsson et al. (23)	Bengtsson et al. 2012	Cohort Study	7 Years	Low	Good	Ac-IgG antibody, CRP	Yes	102	14	277	Middle-aged female SLE patients have an 8 to 9-fold greater risk of cardiovascular events (CVEs) than the general population. Hypertension and the presence of anti-cardiolipin IgG antibodies are significant predictors of CVEs.
Bilgen et al. (24)	Bilgen et al. 2018	Cross-Sectional Study	5 Years	Low	Good	CRP	Yes	N/A	N/A	90	PsA and RA patients showed impaired endothelial function (lower FMD) compared to healthy controls. CIMT was higher in RA patients but not significantly different between PsA patients and healthy controls. PsA's chronic inflammation likely contributes to early atherosclerotic changes.
Borowiec et al. (25)	Borowiec et al. 2021	Cross-Sectional Study	5 Years	Moderate	Good	hsCRP	Yes	71	31	106	Age was the only independent predictor of cardiovascular events.Persistent elevation of inflammatory and prothrombotic markers like hs-CRP and D-dimer was associated with higher cardiovascular risks despite disease remission.
Bultink et al. (26)	Bultink et al. 2005	Cross-sectional study	6.7 Years	Moderate	Good	ADMA, hsCRP, Lupus anticoagulant, Homocysteine, anti-dsdna	Yes	33	8	107	Plasma ADMA levels are significantly associated with cardiovascular events (CVEs) in SLE patients. High ADMA levels correlate with disease activity (e.g., high SLE Disease Activity Index (SLEDAI) scores) and organ damage. ADMA may contribute to accelerated atherosclerosis and CVEs through endothelial dysfunction​
Cioffi et al. (27)	Cioffi et al. 2020	Cross-Sectional Study	3.7 Years	Low	Good	ACPA, hsCRP	Yes	N/A	N/A	241	Left ventricular hypertrophy (LVH) was present in 16% of normotensive, normoglycemic rheumatoid arthritis (RA) patients. LVH was strongly associated with obesity, older age, and ACPA positivity. Patients with LVH had a significantly higher risk of cardiovascular events (31%) compared to those without LVH (11%). LVH independently predicted adverse cardiovascular outcomes.
Davies et al. (28)	Davies et al. 2021	Cross-sectional Study	1 year	Moderate	Good	sVCAM-1, IL-6	Yes	N/A	N/A	227	IL-6 trans-signaling is implicated in vascular dysfunction in RA.Elevated sVCAM-1 is associated with cardiovascular risk and subclinical atherosclerosis progression in RA patients.
Dessein et al. (29)	Dessein et al. 2005	Cohort study	1 Year	Moderate	Good	VCAM-1, ICAM-1, ELAM-1, hs-CRP, IL-1, IL-6, and TNF-α.	Yes	23	5	154	RA patients exhibit higher levels of endothelial dysfunction markers compared to controls. IL-6, rheumatoid factor, and low GFR independently predict endothelial dysfunction. Both traditional and non-traditional cardiovascular risk factors influence endothelial dysfunction in RA. VCAM-1 is linked to carotid atherosclerosis.
Dimitroulas et al. (30)	Dimitroulas et al. 2017	Cross-sectional study	4 Years	Moderate	Good	ADMA, SDMA	Yes	N/A	N/A	186	SDMA and ADMA are significantly associated with vascular dysfunction in RA patients with high systemic inflammation. High inflammatory load exacerbates associations between these biomarkers and microvascular endothelial dysfunction and arterial stiffness. Dimethylarginines may not only be markers of dysfunction but also active contributors to endothelial injury.
Divard et al. (31)	Divard et al. 2017	Cross-sectional study	2.4 Years	Low	Good	Hs-cTnT	Yes	20	N/A	81	HS-cTnT is independently associated with subclinical atherosclerosis in systemic lupus erythematosus (SLE) patients. Detectable HS-cTnT correlates with carotid plaques, even in those classified as low cardiovascular risk.
Elnabawi et al. (32)	Elnabawi et al. 2021	Cross-Sectional Study	1.5 Years	Moderate	Good	CCL20, IL-6, IL-17A, hsCRP	Yes	N/A	N/A	38	CCL20 and IL-6 is strongly associated with vascular endothelial inflammation and may serve as a biomarker for impaired vascular health in psoriasis
Fasano et al. (33)	Fasano et al. 2018	Cohort Study	16 Years	Moderate	Good	Antiphospholipid Antibody (aPL), Cardiolipin IgG–IgM, Beta-2 Glycoprotein-1 IgG–IgM (b2GP-1), Lupus Anticoagulant (LAC)	Yes	101	16	507	Lower incidence of cardiovascular events in Italian SLE patients compared to North European and American cohorts. Risk factors for CV events include hypertension, aPL positivity, and smoking. Use of hydroxychloroquine (HCQ) and aspirin showed a protective effect against cardiovascular events.
Finckh et al. (34)	Finckh et al. 2012	Cohort Study	9 Years	Moderate	Good	C-reactive protein (CRP), anti-cyclic citrullinated peptide (anti-CCP) antibodies, rheumatoid factor (RF), N-terminal pro–brain natriuretic peptide (NT-proBNP), oxidized low-density lipoprotein (ox-LDL), and anti–apolipoprotein A-I (anti–Apo A-I) IgG	Yes	33	16	118	Anti–Apo A-I significantly improved the Framingham 10-year cardiovascular risk score (FRS) predictive ability for MACE, increasing the AUC from 0.72 to 0.81 and the IDI by 175%. NT-proBNP and ox-LDL were significant but did not improve the predictive ability of the FRS.
Gerardino et al. (35)	Gerardino et al. 2019	Cross-Sectional Study	1.9 Years	Moderate	Good	hs-crp, hcy	Yes	39	9	139	Elevated hs-CRP correlates with disease activity, damage accrual, and cardiovascular disease (CVD) risk. Hcy levels correlate with an altered lipid profile but not directly with disease activity or damage accrual in SLE patients
Gheita et al. (36)	Gheita et al. 2012	Cohort Study	5.5 Years	Moderate	Good	hs-CRP, anti-ds-DNA	Yes	N/A	N/A	75	SLE patients had significantly higher hs-CRP levels and increased IMT compared to controls. Positive correlation between hs-CRP levels and IMT, as well as SLEDAI, indicating hs-CRP may be a useful marker for disease activity and subclinical atherosclerosis. Patients with positive anti-ds-DNA had higher hs-CRP levels.
Gullo et al. (37)	Gullo et al. 2014	Cross-sectional study	2 Years	Moderate	Good	hs-CRP, Fibrinogen	Yes	N/A	N/A	50	RA patients had lower CD34+ cell counts and higher ROS, TLR3, and IL-1β levels. Positive correlation between inflammation markers (CRP, IL-1β) and arterial stiffness indices (PWV, AIx). Chronic inflammation in RA with biomarkers like hsCRP and Fibrinogen is associated with impaired vascular repair and accelerated atherosclerosis.
Gustafsson et al. (38)	Gustafsson et al. 2009	Cohort Study	9.5 Years	Moderate	Good	antiphospholipid antibodies (aPL), von Willebrand factor (vWf), and soluble vascular cell adhesion molecule-1 (sVCAM-1), hscrp, fibrinogen, IL6,	Yes	76	N/A	182	Age, positive antiphospholipid antibodies, endothelial cell damage markers, and absence of thrombocytopenia were significant predictors of cardiovascular events (CVE). Activation of the endothelium and coagulation system are pivotal in SLE-related CVD. Subgroups of SLE patients show variable risks for CVEs
Gustafsson et al. (39)	Gustafsson et al. 2012	Cohort Study	12.3 Years	Moderate	Good	hs-CRP, sVCAM-1, aPL and Cystatin-C	Yes	72	7	208	High cystatin C levels and arterial disease are strong predictors of overall mortality. Biomarkers like sVCAM-1, hsCRP, and aPL are significant predictors of CVM. Traditional risk factors, except smoking, are less impactful for CVM in SLE patients.
Huang et al. (40)	Huang et al. 2024	Cohort	4.81 Years	Moderate	Good	ACL, Anti-beta-2gp1, Lupus anticoagulant	Yes	240	41	2399	A predictive model for cardiovascular and cerebrovascular events (CCEs) in patients with systemic lupus erythematosus (SLE) was developed. Significant risk factors include male gender, smoking, hypertension, age at onset >40, and high-dose glucocorticoids. Protective factors include cutaneous involvement, arthritis, and hydroxychloroquine usage. The model's C-index was 0.801, showing strong predictive power.
Huang et al. (41)	Huang et al. 2025	Cohort	13 years	Moderate	Good	Anti-dsDNA, ACL, Anti-beta-2gp1, Lupus Anticoagulant, aPL	Yes	117	27	1573	Lupus anticoagulant (LA) is the strongest biomarker predicting ASCVD in SLE patients, followed by anticardiolipin antibodies (aCL) and anti-β2 glycoprotein I antibodies (aβ2GPI), with IgG isotypes showing stronger associations than IgM. High-risk profiles, including double or triple aPLs positivity, significantly increase cardiovascular risk. Aspirin has a protective effect, reducing ASCVD risk in aPL-positive patients. These findings underscore the importance of targeted monitoring and prevention in high-risk SLE populations.
Icli et al. (42)	Icli et al. 2015	Cross-sectional Study	0.5 Years	Moderate	Good	hsCRP, endocan	Yes	N/A	N/A	88	Higher endocan levels are observed in patients with SLE compared to controls. A positive correlation between endocan levels and cIMT, suggesting endocan's role in subclinical atherosclerosis. Endocan may serve as a biomarker for predicting subclinical atherosclerosis in SLE patients. hsCRP is best biomarker for predicting CVD.
Karpouzas et al. (43)	Karpouzas et al. 2023	Cohort Study	7 Years	Moderate	Good	RF, hs-CRP, anti-β2GPI IgA	Yes	71	25	144	hs-cTnI improves prediction of coronary plaque presence and may guide non-invasive coronary atherosclerosis evaluation. Anti-β2GPI IgA predicts progression to extensive/obstructive plaque in patients with non-extensive/non-obstructive disease, especially when hs-cTnI levels are high.
Karpouzas et al. (44)	Karpouzas et al. 2024	Cohort Study	7 Years	Moderate	Good	RF, hs-CRP, CLC, CAC	Yes	N/A	18	100	Changes in CLC are associated with coronary atherosclerosis progression in rheumatoid arthritis (RA). Prednisone unfavorably affects, while biologics and statins favorably modulate the relationship between CLC and atherosclerosis progression. Novel therapies targeting cholesterol loading in macrophages may complement conventional treatments for cardiovascular risk reduction in RA.
Khairy et al. (45)	Khairy et al. 2016	Cross-Sectional Study	7.9 Years	Low	Good	Leptin, oxidized LDL (oxLDL), homocysteine, triglycerides, HDL cholesterol, lupus anticoagulants.	Yes	29	6	78	SLE patients with CVD had significantly higher levels of leptin, homocysteine, triglycerides, and oxLDL. Higher carotid intima-media thickness (CIMT) was observed in SLE patients with CVD compared to controls. Inflammatory markers (e.g., hsCRP, lupus anticoagulants) were more elevated in SLE with CVD.
Kiani et al. (46)	Kiani et al. 2007	Cohort Study	5 Years	Moderate	Good	Dimethylarginine (ADMA), anti-dsDNA, anti-Sm, anti-RNP, complement levels (C3, C4), and inflammatory markers like ESR and hs-CRP.	Yes	N/A	N/A	200	Elevated ADMA levels are associated with African American ethnicity, anti-dsDNA, low complement, prednisone use, and coronary calcium presence.ADMA identifies SLE patients with normal lipid profiles but at risk for atherosclerosis.
Kobayashi et al. (47)	Kobayashi et al. 2021	Cohort Study	4 Years	Moderate	Good	BMP9, PTX3, TNFRSF11A, PGF, NT-proBNP, ADM, hs-TnT	Yes	170	51	355	Circulating biomarkers are associated with cardiac structural and functional abnormalities in rheumatoid arthritis.Biomarkers like NT-proBNP and hs-TnT indicate myocardial stress and damage.Elevated left ventricular mass index (LVMi) and left atrial volume index (LAVi) predict higher cardiovascular event rates.
Krochin et al. (48)	Krochin et al. 2012	Cross-sectional Study	1 Year	Low	Good	Asymmetric dimethylarginine (ADMA), symmetric dimethylarginine (SDMA), L-arginine, and 8-iso-prostaglandin F2α (8-iso-PGF2α) and CRP	Yes	22	N/A	100	ADMA levels are significantly elevated in RA patients compared to controls. CRP is the independent predictor of elevated ADMA and 8-iso-PGF2α levels. Positive correlation between oxidative stress (8-iso-PGF2α) and inflammation in RA. High disease activity in RA (DAS28 score 5.2 ± 1.1) is associated with increased biomarkers of endothelial dysfunction.
Liakouli et al. (49)	Liakouli et al. 2024	Cross-Sectional Study	2 Years	Low	Good	Biomarkers assessed include carotid and femoral intima-media thickness (cIMT, fIMT), flow-mediated dilatation (FMD), C-reactive protein (CRP)	Yes	190	45	613	Elevated CRP (>0.5 mg/dl) is significantly associated with an increased risk of subclinical atherosclerosis in systemic sclerosis (SSc) patients, highlighting its role as a predictive inflammatory biomarker for cardiovascular disease (CVD) risk. High CRP levels reflect systemic inflammation, which contributes to endothelial dysfunction and the development of atherosclerosis. Addressing inflammation may serve as a modifiable strategy to reduce cardiovascular events in SSc patients.
Lopez-Longo et al. (50)	Lopez-Longo et al. 2009	Cohort Study	15 Years	Low	Good	Anti-CCP	Yes	157	92	937	Positive anti-CCP antibodies are associated with an increased risk of ischemic heart disease (IHD) in RA patients (OR 2.58, 95% CI 1.17–5.65). Anti-CCP-positive patients had a higher global mortality rate than anti-CCP-negative patients (11.2% vs. 6.8%).There is no association between anti-CCP antibodies and traditional cardiovascular risk factors like smoking, diabetes, or hypertension.
Mackey et al. (51)	Mackey et al. 2015	Cohort Study	10 Years	Low	Good	Anti-CCP (Anti-Cyclic Citrullinated Peptide), RF (Rheumatoid Factor), WBC (White Blood Cell count), and IL-6.	Yes	N/A	N/A	9988	Postmenopausal women with RA have a 1.5- to 2.5-fold higher risk of cardiovascular outcomes, including CHD, stroke, and CVD mortality. Inflammation markers (WBC count, IL-6) and joint pain severity were strongly associated with CVD outcomes. Anti-CCP and RF were not significantly associated with higher CVD morbidity or mortality​
Mcmahon et al. (52)	Mcmahon et Al. 2014	Cohort Study	2.5 Years	Moderate	Good	Proinflammatory HDL (piHDL), leptin, plasma soluble TWEAK (sTWEAK), homocysteine levels, hsCRP	Yes	25	11	310	A high-risk PREDICTS profile combining biomarkers and traditional risk factors predicted increased odds of carotid plaque presence and progression in SLE patients.Patients with a high-risk profile had a 28-fold increased odds of plaque presence.Age ≥48 years, high piHDL function, leptin levels ≥34 ng/dl, and high sTWEAK levels were strong predictors of carotid plaque.
Mercado et al. (53)	Mercado et al. 2017	Cross-sectional Study	1 Year	Moderate	Good	Carotid to femoral pulse wave velocity (cfPWV), serum lipids (total cholesterol, LDL-c, HDL-c, triglycerides), C-reactive protein (CRP), erythrocyte sedimentation rate (ESR), rheumatoid factor (RF), anti-cyclic citrullinated peptide (Anti-CCP)	Yes	N/A	N/A	106	RA disease duration is a predictor of increased vascular stiffness (cfPWV). Each year of RA contributes more to vascular stiffness than aging without RA. Patients with RA duration ≥10 years exhibited higher cfPWV compared to those with shorter disease duration. Age, disease duration, and triglycerides were significant predictors of cfPWV.
Mok et al. (36)	Mok et al. 2013	Cohort Study	0.3 Years	Moderate	Good	hscrp	Yes	20	5	289	hsCRP is detectable in 77% of patients with active systemic lupus erythematosus (SLE).hsCRP levels correlate with SLE disease activity, especially serositis, musculoskeletal, and hematological disease.Elevated hsCRP is associated with male sex, chronic smoking, diabetes mellitus, and a history of arterial thrombosis.
Mongin et al. (54)	Mongin et al. 2024	Cohort Study	4.4 Years	Moderate	Good	Anti-apolipoprotein A-1 IgG, hsCRP, NT-ProBNP, Hs-cTnT, RF	Yes	546	136	1472	Anti-apolipoprotein A-1 IgG independently predicts CV deaths and has limited prediction capability for MACE. Associated with the lipid paradox in RA, indicating potential underestimation of CV risk in RA patients
Nasr et al. (55)	Nasr et al. 2021	Cross-Sectional Study	1 Year	Moderate	Good	hsCRP, anti-dsDNA, Hs-TnT, CKMB	Yes	N/A	N/A	50	Hs-TnT is a sensitive marker of myocardial injury in lupus nephritis. CKMB lacks sensitivity for early myocardial injury detection in lupus nephritis patients. Lupus nephritis is associated with higher prevalence and severity of subclinical cardiovascular disease compared to systemic lupus erythematosus alone.
Nikiphorou et al. (56)	Nikiphorou et al. 2019	Case-control	13 years	Moderate	Good	CRP, RF, anti-CCP	Yes	N/A	691	13182	RA patients have excess cardiovascular risk before and after diagnosis. Stroke and heart failure are more prevalent at RA diagnosis. Traditional risk factors do not fully explain the increased CVD risk in RA patients.
Nowak et al. (57)	Nowak et al. 2016	Cross-Sectional Study	1 Year	Moderate	Good	Anti-CCP antibodies.Oxidized LDL (oxLDL). Anti-oxLDL antibodies. Erythrocyte Sedimentation Rate (ESR).	Yes	14	N/A	61	SCORE and oxLDL were elevated in RA patients compared to controls. Anti-CCP positivity correlated with increased carotid IMT and SCORE. Disease duration and ESR significantly influenced cardiovascular (CV) risk. Anti-oxLDL antibodies showed a protective role against CV risk in RA patients.
Pàmies et al. (58)	Pàmies et al. 2024	Cross-sectional Study	4 Years	Low	Good	CRP, fibrinogen, RF	Yes	118	23	199	Elevated Catestatin (CST) levels were associated with RF and ACPA positivity. Inverse associations were observed between Fetuin-A levels and inflammation markers like ESR and fibrinogen, suggesting a protective role​
Parker et al. (59)	Parker et al. 2014	Cross-Sectional Study	0.5 years	Low	Good	hsCRP, VCAM-1, VEGF, and sEPCR.	Yes	12	2	49	SLE patients had elevated endothelial microparticles and impaired flow-mediated dilatation. Improved disease control was associated with significant reductions in EMP levels and improvements in endothelial function.
Perna et al. (60)	Perna et al. 2010	Cross-sectional Study	12 Years	Moderate	Good	Crp, ADMA, Homocysteine, Anti-phospholipid antibodies	Yes	36	3	125	ADMA and homocysteine levels are biomarkers of arterial stiffness in SLE. These biomarkers are not correlated with carotid atherosclerosis. Arterial stiffness is associated with older age at SLE diagnosis, disease duration, and diabetes mellitus.
Robinson et al. (61)	Robinson et al. 2019	Cross-Sectional Study	1.3 Years	Moderate	Good	CRP, Lupus AnticoagulantApoB:ApoA1 ratio, lipid particles (HDL, LDL, VLDL), and atherogenic indices	Yes	N/A	N/A	62	ApoB:ApoA1 ratio is a significant biomarker for CV risk in JSLE. Stratification of patients can help in tailored therapeutic interventions, including lipid-modifying therapies.
Rodrigues et al. (63)	Rodrigues et al. 2020	Cross-Sectional Study	3 Years	Moderate	Good	NT-proBNP, high-sensitivity troponin T (hsTnT), C-reactive protein (CRP), and erythrocyte sedimentation rate (ESR)	Yes	43	12	319	Subclinical ventricular dysfunction was prevalent in 17% of RA patients without known cardiovascular disease. Age was the strongest predictor of ventricular dysfunction. NT-proBNP levels were higher in patients with ventricular dysfunction. Diastolic dysfunction prevalence was lower using 2016 guidelines compared to 2009 guidelines.
Roghan et al. (64)	Roghan et al. 2024	Cross-sectional Study	0.7 Months	Moderate	Good	**IL-6**, **NT-proBNP**, **HS-CRP**, and **CXCL9**	Yes	N/A	N/A	60	IL-6 positively correlates with cardiovascular risk predictors (FRS, SCORE) and biomarkers (NT-proBNP, HS-CRP). IL-6 levels are higher in RA patients compared to controls and correlate with disease activity (DAS-28). IL-6 could be considered a biomarker for subclinical cardiovascular risk in RA patients.
Roman et al. (65)	Roman et al. 2005	Cross-sectional study	1 Year	Moderate	Good	CRP and IL-6	Yes	19	3	286	Arterial stiffness is significantly increased in patients with chronic inflammatory diseases compared to controls, independent of atherosclerosis. Increased arterial stiffness is associated with disease duration, cholesterol levels, CRP, and IL-6. The findings suggest inflammation, rather than structural changes, as a critical factor in vascular stiffness.
Ruscitti et al. (66)	Ruscitti et al. 2017	Cross-sectional study	1 Year	Moderate	Good	RF, ACPA, CRP, ESR	Yes	538	154	1176	Low prevalence of subclinical atherosclerosis (16%) and history of cardiovascular events (6.9%) compared to global reports. High disease activity (DAS28 >5.1) associated with both subclinical atherosclerosis and CV events. Traditional CV risk factors (metabolic syndrome, hypertension, diabetes) were significantly associated with elevated CRP.
Ruscitti et al. (67)	Ruscitti et al. 2019	Cross-sectional study	3 Years	Moderate	Good	hsCRP	Yes	98	400	841	Increased prevalence and incidence of subclinical and clinical atherosclerosis in RA, particularly in participants with a disease duration of <5 years. Remission reduces the risk of subclinical and clinical atherosclerosis. T2D, HBP, ACPA positivity, and elevated CRP levels are predictive factors for increased atherosclerosis risk.
Rydell et al. (68)	Rydell et al. 2023	Cohort Study	24 Years	Moderate	Good	Anti-CCP, CRP	Yes	66	14	233	High levels of COMP may predict increased risk of CVD and coronary artery disease (CAD) in RA. Persistent disease activity over two years significantly increases the risk of CVD/CAD. Traditional risk factors like age, sex, hypertension, and diabetes remain significant predictors.
Saadany et al. (69)	Saadany et al. 2011	Cross-sectional Study	1 Years	Moderate	Good	ADMA, CRP, MCP-1	Yes	N/A	N/A	50	SLE patients show higher levels of ADMA, hs-CRP, and MCP-1 than controls, which are significantly correlated with increased carotid intima-media thickness (IMT). MCP-1 A−2518G polymorphism, particularly the G/G genotype, is associated with a higher risk of carotid atherosclerosis. ADMA and MCP-1 are strong independent predictors of increased IMT.
Sandoo et al. (70)	Sandoo et al. 2014	Cohort Study	6 Years	Moderate	Good	ADMA, CRP	Yes	132	21	201	Cumulative inflammatory burden (measured by ESR and CRP) is positively associated with ADMA levels in RA patients. This association is independent of classical cardiovascular disease (CVD) risk factors. The findings suggest that chronic systemic inflammation may contribute to endothelial dysfunction in RA patients.
Santos et al. (71)	Santos et al. 2012	Cross-Sectional Study	1.6 Years	Moderate	Good	Soluble intercellular adhesion molecule-1 (sICAM-1), vascular cell adhesion molecule-1 (sVCAM-1), thrombomodulin (TM), and tissue factor (TF).	Yes	77	13	358	Women with SLE displayed distinct vascular biomarker profiles compared to those with RA. SLE patients had higher sICAM-1 and TM levels and lower TF levels than RA patients. Early vascular alterations were more pronounced in SLE patients, aligning with their higher cardiovascular (CV) risk.
Sari et al. (72)	Sari et al. 2009	Cross-Sectional Study	0.8 Years	Moderate	Good	ADMA, ET-1, hsCRP, ESR	Yes	N/A	N/A	86	Increased ADMA levels suggest impaired nitric oxide metabolism in ankylosing spondylitis (AS) patients. Anti-TNF-α treatments showed beneficial effects on vascular function. No difference in ET-1 levels between AS patients and controls.
Sentruk et al. (73)	Sentruk et al. 2016	Cohort Study	11 years	Moderate	Good	ADMA, CRP, ESR, RF, Anti-CCP	Yes	N/A	N/A	69	ADMA levels are significantly higher in RA patients than in the control group. FMD is negatively correlated with ADMA and disease duration. RA patients with longer disease durations have increased cardiovascular risk. ADMA is a potential marker for cardiovascular risk assessment in RA patients.
Skaggs et al. (74)	Skaggs et al. 2021	Cohort Study	10 Years	Moderate	Good	HDL, Leptin, Homocysteine, sTWEAK, hsCRP	Yes	205	90	497	High PREDICTS score strongly predicts cardiovascular events in SLE patients. Hypertension and high biomarker levels are key risk factors. SLE patients face a 4.2-fold increased hazard ratio for cardiovascular events compared to controls.
Sodergren et al. (75)	Sodergren et al. 2019	Cohort Study	5 Years	Moderate	Good	IL-6, MIF, MPO, pentraxin3, IL-18, MIC-1, TNF-R2, ICAM-1, and VCAM-1.	Yes	N/A	N/A	111	RA disease affects biomarkers like IL-18, MIC-1, and ICAM-1, linking RA with subclinical atherosclerosis. Elevated levels of these biomarkers may indicate increased cardiovascular disease (CVD) risk in RA patients
Solow et al. (76)	Solow et al. 2015	Cross-Sectional Study	5 Years	Moderate	Good	IL-4, TNF-α, anti-citrullinated ApoE, anti-citrullinated vimentin, anti-citrullinated fibrinogen, CRP.	Yes	67	38	906	Vascular calcifications were independently associated with increased all-cause mortality in RA patients. IL-4 and anti-citrullinated ApoE levels were significantly elevated in patients with vascular calcifications. Prednisone use and diabetes were positively associated with vascular calcifications.
Svenungsson et al. (77)	Svenungsson et al. 2001	Case-Control Study	29 Years	Moderate	Good	Hcy, Anti-dsDNA, Fibrinogen, hsCRP, Lupus anticoagulant	Yes	N/A	3	78	SLE cases with CVD had increased IMT (intima-media thickness), dyslipidemia, higher levels of OxLDL, and lupus anticoagulant compared to controls. There were significant differences in inflammatory markers and other biomarkers between cases and controls.
Tam et al. (78)	Tam et al. 2025	Cross-Sectional Study	1 Year	Moderate	Good	flow-mediated dilation (FMD), von Willebrand factor (vWF), plasminogen activator inhibitor-1 (PAI-1), fibrinogen binding, beta-thromboglobulin (ß-TG), and P-selectin expression	Yes	N/A	N/A	27	Acute hyperhomocysteinemia increased markers of endothelial dysfunction (vWF) and platelet activation (fibrinogen binding) in SLE patients. PAI-1 levels increased significantly only in controls. FMD was not significantly impaired post-loading in either group.
Trives et al. (79)	Trives et al. 2021	Cross-sectional Study	9 Years	Moderate	Good	hsCRP, anti-dsDNA, Anti-β2GP IgG Ab, Apo A, Apo B, IL6	Yes	N/A	N/A	80	Anti-dsDNA antibody positivity and persistence in SLE patients are linked to endothelial dysfunction, proatherogenic dyslipidemia, and accelerated atherosclerosis—with monocytes showing distinct inflammatory profiles and neutrophils exhibiting enhanced NETosis.*in vitro*, Ig-dsDNA triggers monocyte apoptosis and endothelial activation via Fc receptor binding, underscoring its role in driving a coordinated immune and vascular activation that heightens cardiovascular risk.
Turiel et al. (80)	Turiel et al. 2009	Case-control study	1.6 Years	Moderate	Good	Anti-CCP, Homocysteine, hsCRP, ADMA	Yes	N/A	N/A	50	Early RA patients had significantly higher plasma ADMA levels, reduced coronary flow reserve, and increased carotid intima-media thickness compared to controls. Moreover, higher ADMA levels were strongly and negatively correlated with CFR, implicating ADMA in early endothelial dysfunction and atherosclerosis.
Veeranna et al. (81)	Veeranna et al. 2011	Cohort Study	6 Years	Moderate	Good	hsCRP, Homocysteine	Yes	2868	792	3660	Elevated hsCRP and Hcy is significantly associated with increased risk of CVD and CHD events. Addition of Hcy to the Framingham Risk Score (FRS) significantly improved risk stratification, especially in intermediate-risk populations
Vuilleumier et al. (82)	Vuilleumier et al. 2010	Cohort Study	15 Years	Moderate	Good	Anti-apolipoprotein A-1 IgG, IL-6, TNF-alpha, CRP	Yes	43	16	133	Anti-Apo A-1 IgG is an independent predictor of major cardiovascular events in rheumatoid arthritis (RA). Positivity for anti-Apo A-1 IgG is associated with higher levels of IL-8, oxidized LDL, and MMP-9. Anti-Apo A-1 IgG induces pro-inflammatory responses in macrophages.
Wahab et al. (83)	Wahab et al. 2015	Cross Sectional Study	1 Year	Moderate	Good	Anti-CCP, HOMA2IR, hsCRP, ESR,	Yes	N/A	N/A	75	RA patients exhibited significantly higher IMT (0.57 ± 0.051 mm) and lower FMD percentages compared to controls. Higher levels of IR and anti-CCP were positively correlated with increased IMT and negatively correlated with FMD.
Wingren et al. (84)	Wingren et al. 2018	Cohort Study	10 Years	Moderate	Good	IL-6, MCP-1	Yes	157	6	737	SLE patients had elevated apoptosis and tissue degradation biomarkers compared to controls. TRAIL receptor 2 is significantly associated with CVD in SLE after adjusting for Framingham risk factors. The association between inflammation biomarkers and CVD was not significant.

In addition, it was possible to perform a meta-analysis to calculate the joint results of the hs-CRP studies predicting incident CVD among adults diagnosed with autoimmune disease without a prior CVD history or symptoms. The confounder factors that were mostly used for adjustment were age, gender, race and smoking status or traditional cardiovascular risk factors such as hypertension, dyslipidemia and diabetes mellitus. The systematic review summarized in Table 1 highlights that numerous studies have investigated a variety of biomarkers for predicting CVD in autoimmune patients, yet none have emerged as definitively predictive. For instance, Aiewruengsurat et al. (17) found only weak correlations between cardiac biomarkers—such as hsTropT and NT-proBNP—and cardiac structure and function in rheumatoid arthritis (RA) patients, while Ajeganova et al. (18) demonstrated that RF or Anti-CCP positivity combined with high inflammatory markers like C-Reactive Protien (CRP) and Erythrocyte Sedimentation Rate (ESR) increased CVD risk, particularly in RA patients with early disease onset. Similarly, Arnab et al. (19) reported that anti-CCP antibodies were associated with markers of atherosclerosis and cardiac dysfunction, and Bakry et al. (20) underscored the role of cytokines, including IL-1β, IL-6, and IL-18, in the risk of ischemic heart disease. Additionally, Bana et al. (21) and Barbarroja et al. (22) provided evidence that elevated levels of inflammatory and fibrosis markers, such as TNF-α and anti-CCP titers, were linked with proatherogenic profiles in SLE and RA patients, respectively. Other studies evaluated biomarkers like ADMA, Symmetric Dimethylarginine (SDMA), and sVCAM-1, which showed associations with endothelial dysfunction, arterial stiffness, and myocardial stress, though these relationships were generally modest. Collectively, while many of these studies suggest that individual biomarkers or panels of markers—ranging from inflammatory cytokines to autoantibodies—can indicate an increased cardiovascular risk, the overall evidence indicates that they are not yet definitive predictors of CVD in autoimmune populations.

### 3.2 Meta-analysis

#### 3.2.1 Analysis of hsCRP as a biomarker for incident CVD

**Figure 2:**
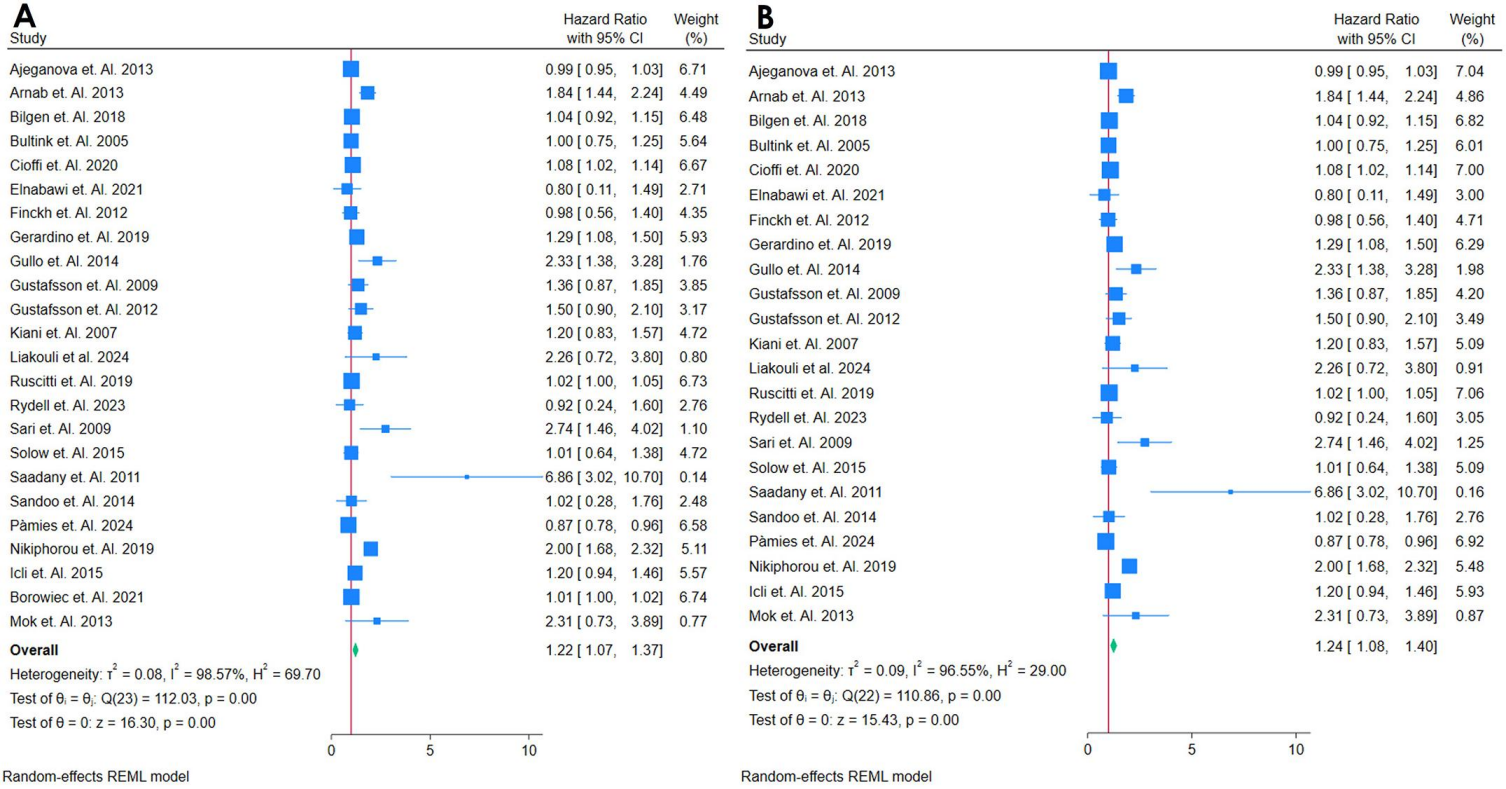
**(A)** Forest plot of studies for hsCRP Biomarker prediction of CVD among adults having Autoimmune diseases without a prior CVD history or symptom. **(B)** Forest plot of Sensitivity Analysis of studies for hsCRP Biomarker prediction of CVD among adults having Autoimmune diseases without a prior CVD history or symptom.

A total of 24 studies assesed the association between hs-CRP and Cardiovascular outcomes among autoimmune patients (Table 1) (18, 19, 21, 24, 26, 27, 32, 34–39, 42, 46, 49, 56, 58, 66, 68–70, 72, 76). All 24 studies showed that hs-CRP was related to higher risk of CVD or incident of mortality. The ability of hs-CRP to predict incident CVD among middle-aged adults without a prior CVD history or symptoms was computed by performing a meta-analysis. The meta-analysis included 24 studies that were found with comparable continuous analysis, using an adjusted hazard ratio per increment of 1 SD unit of the continuous predictor variable (Figure 2A). The meta-analysis for the predictive value of hs-CRP in incident CVD among adults with autoimmune diseases initially demonstrated high heterogeneity [*Q*(22) = 110.86, *p* = 0.00; *I*^2^ = 96.55%; *τ*^2^ = 0.09; *H*^2^ = 29.00], prompting the use of a random-effects model. Despite this variability, the overall effect was highly significant (*z* = 15.43, *p* = 0.00). A funnel plot suggested possible publication bias, which was supported by a significant Egger's test (*p* = 0.03), indicating that smaller studies with null or negative findings may be underrepresented (Supplementary Figure S1). To assess the robustness of these results, a sensitivity analysis was performed by removing the study with the highest weight. While heterogeneity remained elevated [*Q*(23) = 112.03, *p* = 0.00; *I*^2^ = 98.57%; *τ*^2^ = 0.08; *H*^2^ = 69.70], the pooled effect remained statistically significant (*z* = 16.30, *p* = 0.00), reinforcing the main findings (Figure 2B), Funnel Plot is in Supplementary Figure S2. Nevertheless, the presence of substantial heterogeneity and signs of publication bias warrant cautious interpretation and underscore the need for further investigation into potential sources of variability.

#### 3.2.2 Analysis of RF as a biomarker for incident CVD

**Figure 3:**
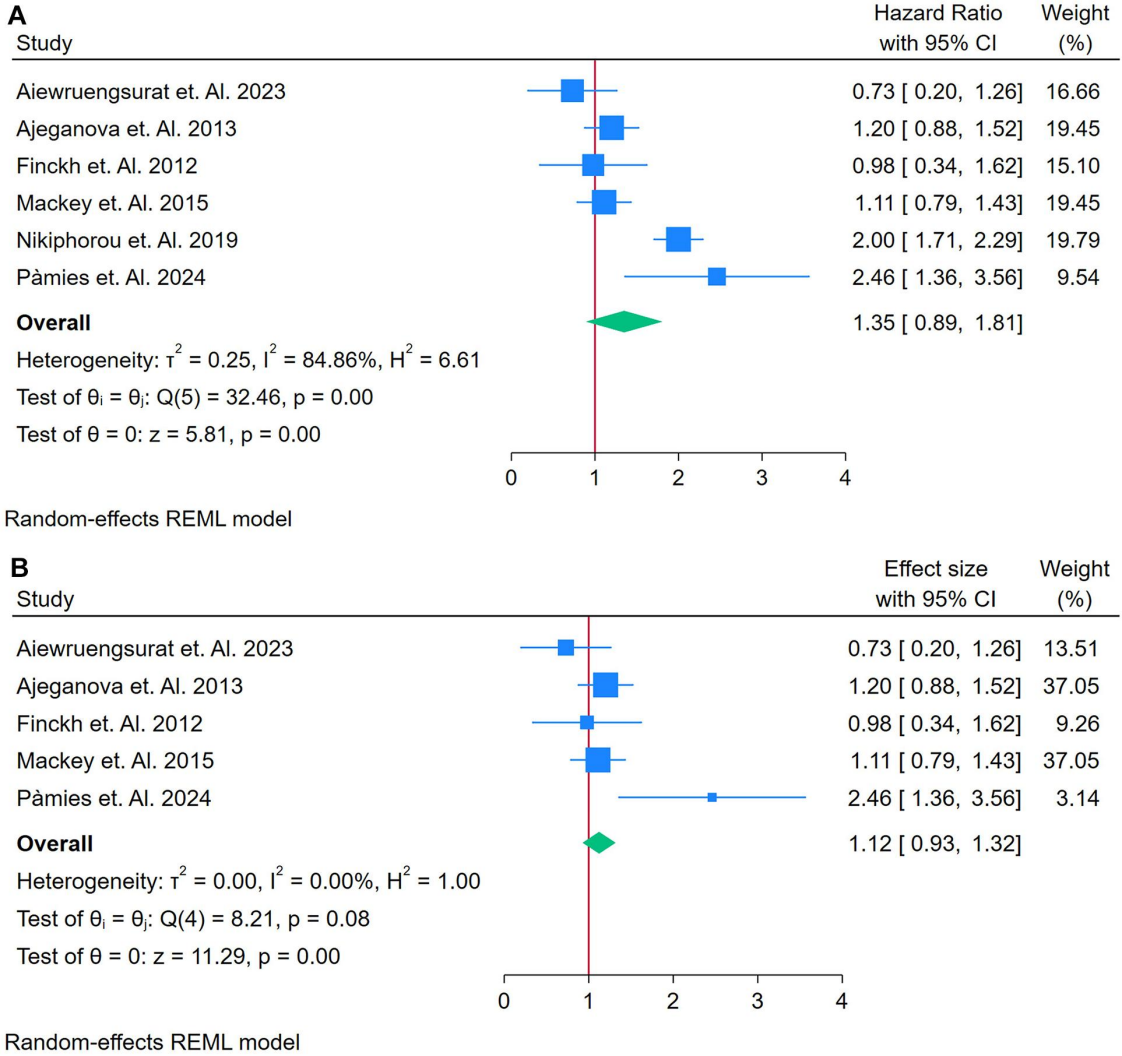
**(A)** Forest plot of studies for RF prediction biomarker of CVD among adults having autoimmune diseases without a prior CVD history or symptom. **(B)** Forest plot of Sensitivity Analysis studies for RF prediction Biomarker of CVD among adults having Autoimmune diseases without a prior CVD history or symptom.

A total of 6 Studies were asses for the association between RF and Cardiovascular Outcomes among autoimmune patients (17, 18, 34, 51, 56, 58). Figure 3A displays a forest plot summarizing six studies that examined the association between RF and incident CVD in adults with autoimmune conditions. Utilizing a random-effects model (REML), the pooled HR was 1.35 (95% CI: 1.08–1.69, *p* < 0.01), indicating a 35% increased risk of developing CVD with elevated RF levels. However, heterogeneity was significant, as demonstrated by Cochran's *Q* test [*Q*(5) = 32.46, *p* = 0.00] and an *I*^2^ value of 84.86% (*τ*^2^ = 0.25, *H*^2^ = 6.61), suggesting that most of the variation in effect sizes stemmed from true differences among studies rather than chance Figure 3A. The overall effect was statistically robust (*z* = 5.81, *p* = 0.00), although the funnel plot (Supplementary Figure S3) revealed potential asymmetry, hinting at possible publication bias or the influence of outlier studies.

To assess the impact of potential outliers, a sensitivity analysis was performed by excluding the study with the highest weight (Figure 3B). This removal reduced the analysis to five studies and substantially decreased heterogeneity [*Q*(4) = 8.21, *p* = 0.08; *I*^2^ = 0.00%, *τ*^2^ = 0.00, *H*^2^ = 1.00]. Under these revised conditions, the pooled HR was attenuated to 1.12 (95% CI: 0.93–1.33) and the effect became borderline or non-significant (*z* = 2.19, *p* = 0.03). The corresponding funnel plot (Supplementary Figure S4) appeared more symmetrical, indicating that the removed study had a disproportionate influence on both the overall effect size and the initial heterogeneity.

In detail, the initial results suggest that elevated RF may predict an increased risk of incident CVD in autoimmune populations, as evidenced by a significant HR of 1.35. However, the high heterogeneity (*I*^2^ = 84.86%) raises concerns about the consistency of this association across studies, implying that differences in study design, population characteristics, or measurement techniques might be driving the effect (Figure 3B). The sensitivity analysis further reveals that the predictive strength of RF is largely dependent on a single, heavily weighted study. When this study is excluded, the association weakens (HR = 1.12) and loses clear statistical significance. These findings indicate that while there is an initial association between RF and CVD risk, its utility as a strong and consistent predictive biomarker is limited, warranting further research with more homogeneous data to better define its predictive value.

#### 3.2.3 Analysis of anti-CCP as a biomarker for incident CVD

**Figure 4:**
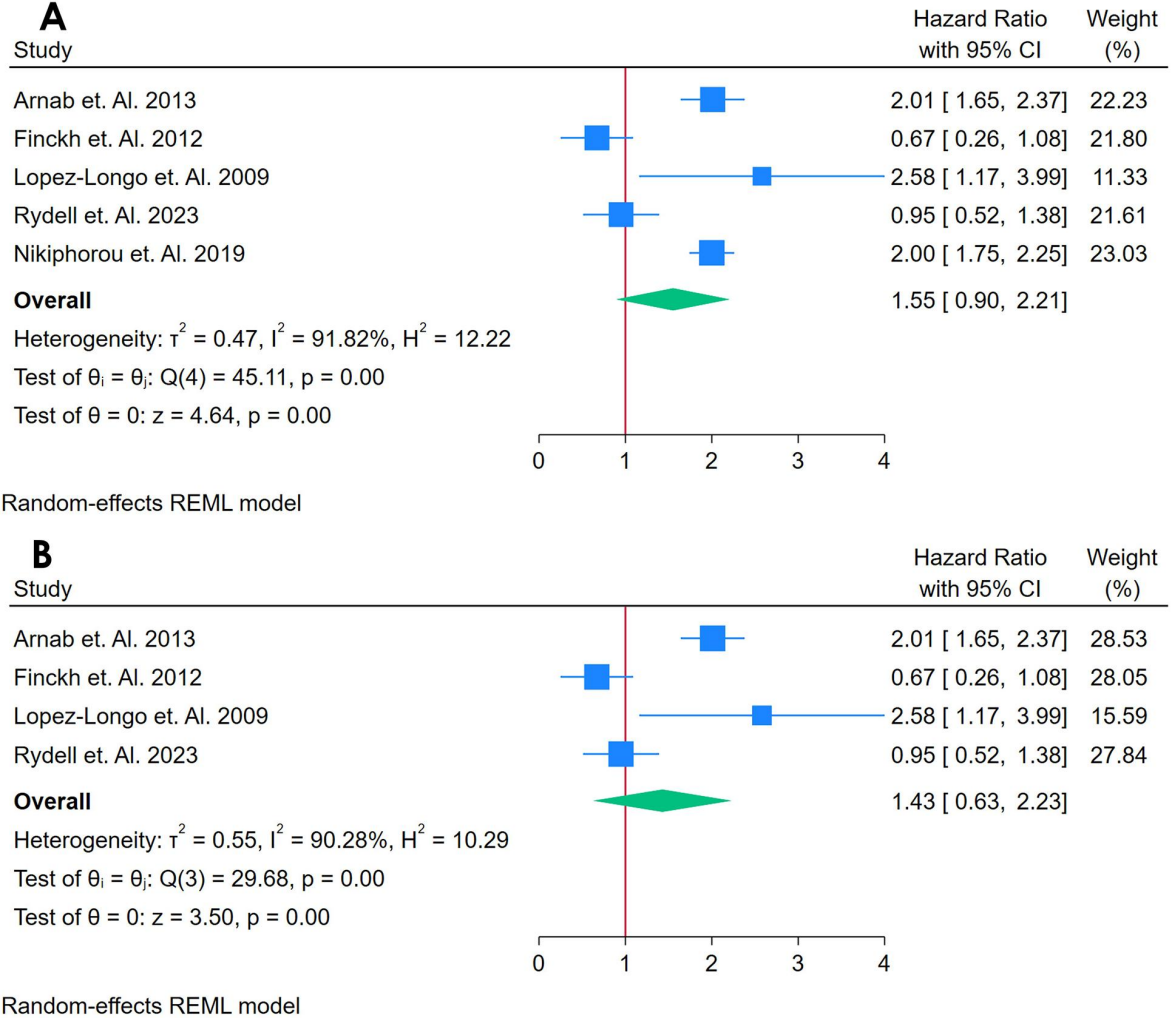
**(A)** Forest plot of studies for anti-CCP biomarker prediction of CVD among adults having autoimmune diseases without a prior CVD history or symptom. **(B)** Forest plot of Sensitivity Analysis of studies for Anti-CCP Biomarker prediction of CVD among adults having Autoimmune diseases without a prior CVD history or symptom.

A total of 5 Studies were asses for the association between RF and Cardiovascular Outcomes among autoimmune patients (19, 34, 50, 56, 68). Figure 4A presents a forest plot of four studies examining the association between Anti-CCP and CVD in adults with autoimmune conditions. Using a REML, the pooled HR was 1.55 (95% CI: 0.90–2.21, *p* < 0.01), indicating a 59% increased risk of CVD in individuals with elevated anti-CCP. Heterogeneity was evaluated using Cochran's *Q* [*Q*(4) = 45.11, *p* = 0.00] and found to be substantial (*I*^2^ = 91.82%, *τ*^2^ = 0.47, *H*^2^ = 12.22), suggesting that most variability in effect sizes stemmed from genuine differences across studies rather than random error. The test of the overall effect was statistically significant (*z* = 4.64, *p* = 0.00). However, the funnel plot (Supplementary Figure S5) showed possible asymmetry, raising concerns about publication bias or an outlier study influencing the pooled result.

A sensitivity analysis (Figure 4B) was conducted by removing the study with the highest weight, reducing the dataset to three studies. This exclusion slightly lowered heterogeneity [*Q*(3) = 29.68, *p* = 0.00; *I*^2^ = 90.28%, *τ*^2^ = 0.00, *H*^2^ = 1.20] and maintained a statistically significant pooled effect (*z* = 5.23, *p* = 0.00). The corresponding funnel plot (Supplementary Figure S6) appeared more symmetrical, indicating that the omitted study had a notable impact on the overall estimate. Despite this change, heterogeneity remained high, highlighting the variability among the remaining studies. Collectively, these findings suggest that anti-CCP may be associated with an increased risk of incident CVD in autoimmune populations, but the pronounced heterogeneity underscores the need for caution in interpreting the pooled effect and emphasizes the importance of additional research to elucidate potential sources of variation.

#### 3.2.4 Analysis of IL-6 as a biomarker for incident CVD

**Figure 5:**
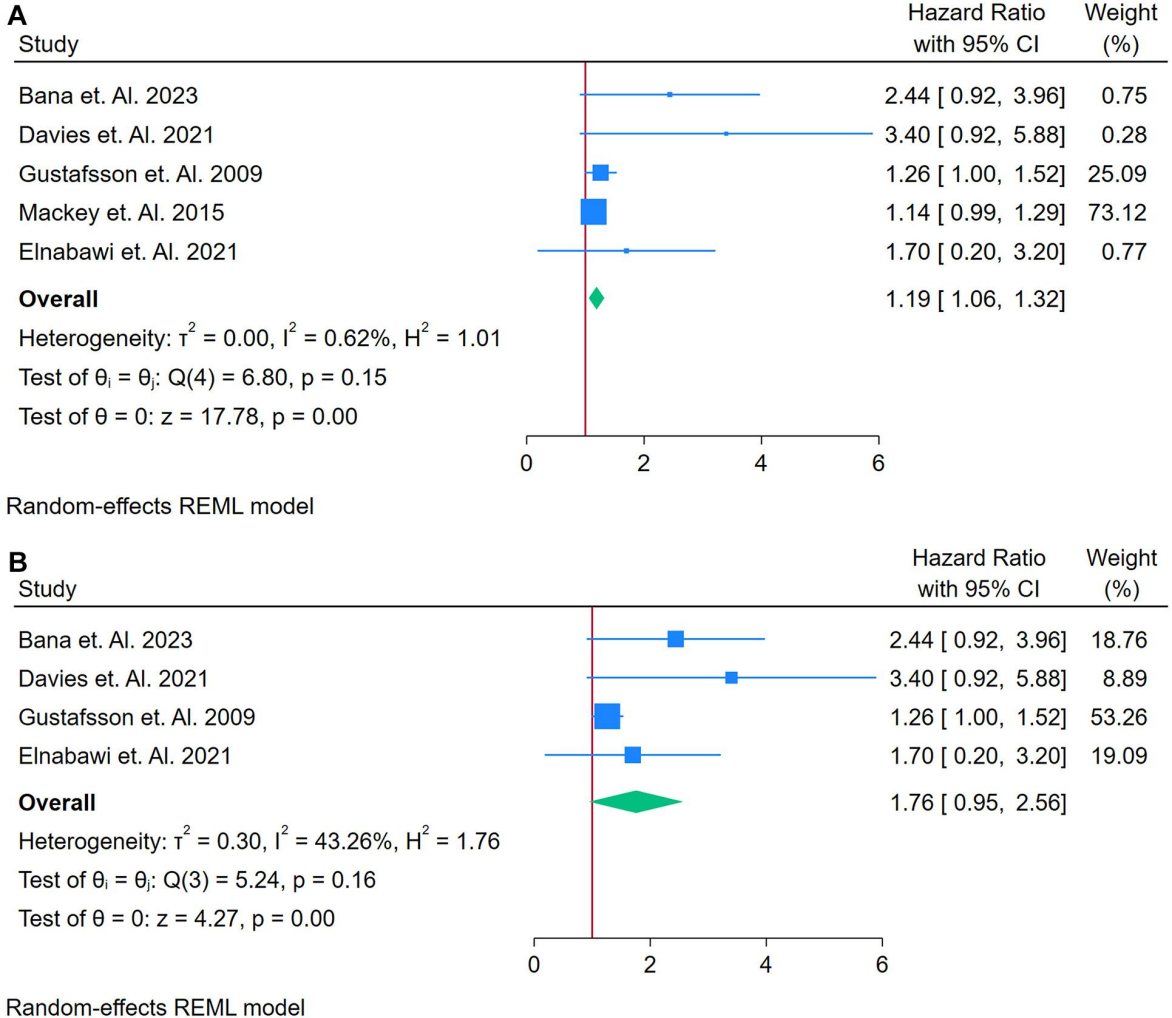
**(A)** Forest plot of studies for IL6 biomarker prediction of CVD among adults having autoimmune diseases without a prior CVD history or symptom. **(B)** Forest plot of studies for sensitivity analysis of IL6 Biomarker prediction of CVD among adults having Autoimmune diseases without a prior CVD history or symptom.

A total of 5 Studies were asses for the association between RF and Cardiovascular Outcomes among autoimmune patients (21, 28, 32, 38, 57). Figure 5A displays a forest plot of five studies evaluating the association between IL-6 and incident CVD among adults with autoimmune conditions. Using a random-effects REML model, the pooled HR was 1.19 (95% CI: 1.06–1.32), suggesting that elevated IL-6 levels were linked to a 19% increased risk of developing CVD. Heterogeneity was minimal, as indicated by Cochran's *Q* [*Q*(4) = 6.49, *p* = 0.16] and low *I*^2^ (0.62%), along with *τ*^2^ = 0.00 and *H*^2^ = 1.01. These statistics imply that the included studies produced largely consistent estimates. The funnel plot (Supplementary Figure S7) appears relatively symmetrical, indicating no obvious publication bias or outlier effects in this initial analysis.

A sensitivity analysis was conducted by removing a potentially influential study (Figure 5B). Under these conditions, the pooled HR increased to 1.76 (95% CI: 0.95–2.56), while heterogeneity rose to moderate levels [*Q*(3) = 29.58, *p* = 0.16; *I*^2^ = 43.26%; *τ*^2^ = 0.30; *H*^2^ = 1.76]. Although the test of the overall effect remained statistically significant (*z* = 4.27, *p* = 0.00), the confidence interval now narrowly encompasses 1, suggesting a less robust association. The updated funnel plot (Supplementary Figure S8) remains mostly symmetrical, implying that publication bias alone does not account for the observed findings. Nonetheless, the shift in effect size and heterogeneity highlights the impact of individual studies on the pooled result, underscoring the need for cautious interpretation and further research to clarify IL-6's predictive role in CVD among individuals with autoimmune disease.

#### 3.2.5 Analysis of LA as a biomarker for incident CVD

**Figure 6:**
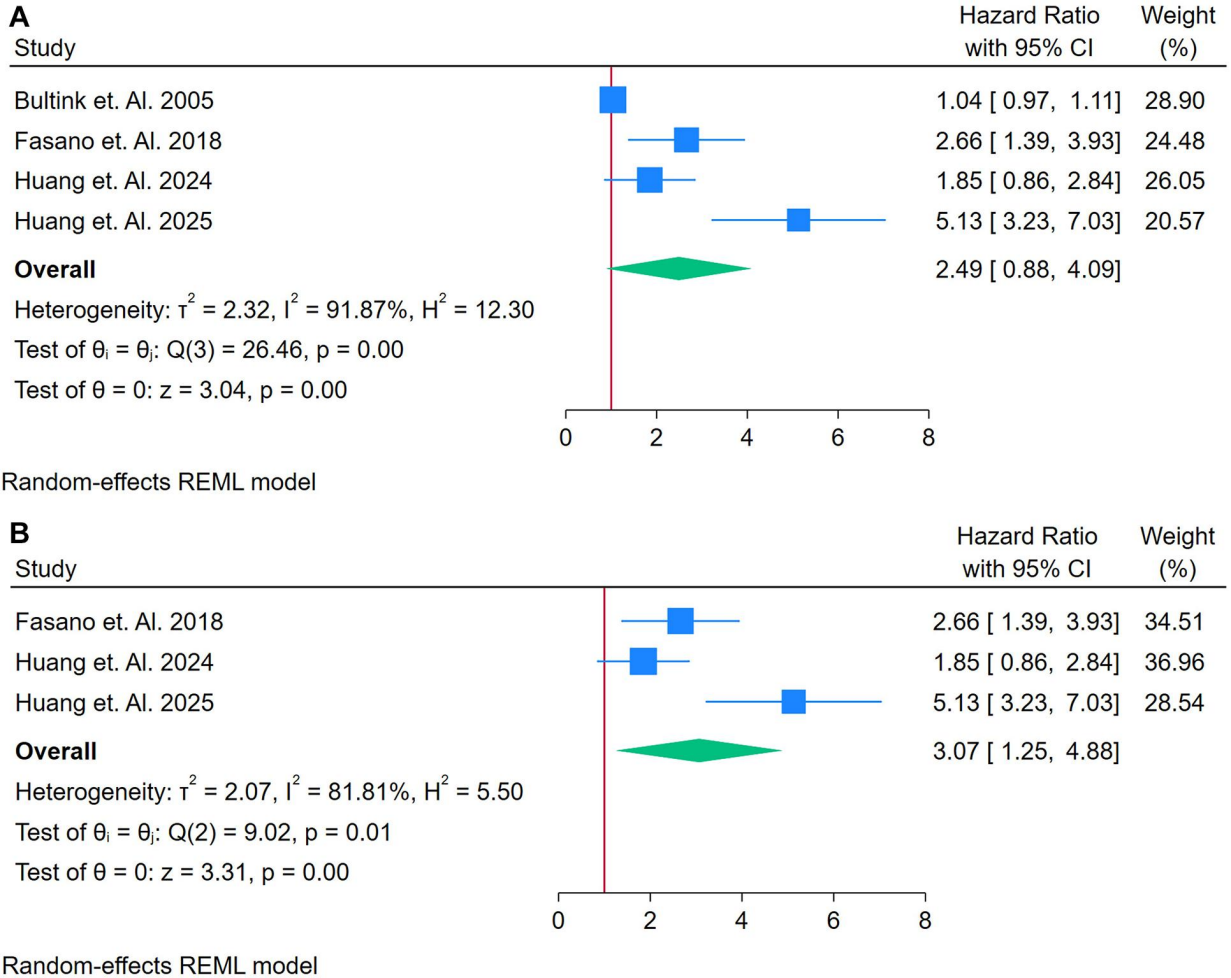
**(A)** Forest plot of studies for lupus anti-coagulant biomarker prediction of CVD among adults having autoimmune diseases without a prior CVD history or symptom. **(B)** Forest plot of sensitivity analysis of studies for Lupus Anti-Coagulant Biomarker prediction of CVD among adults having Autoimmune diseases without a prior CVD history or symptom.

A total of 4 studies were assessed (26, 33, 40, 41). Figure 6A shows a forest plot of four studies assessing the association between LA and incident CVD among adults with autoimmune conditions. Employing a random-effects REML model, the pooled HR was 2.49 (95% CI: 1.80–3.42), indicating a more than twofold increased risk of CVD for individuals testing positive for LA. Heterogeneity was considerable, as evidenced by Cochran's *Q* [*Q*(3) = 26.46, *p* = 0.00] and an *I*^2^ of 91.87% (*τ*^2^ = 2.32, *H*^2^ = 10.23), suggesting that true differences across studies, rather than random error, contributed substantially to the variability. The test of overall effect was statistically significant (*z* = 3.04, *p* = 0.00). Inspection of the funnel plot (Supplementary Figure S9) indicates potential asymmetry, which may point to publication bias or a disproportionately influential study. A sensitivity analysis (Figure 6B) was performed by removing the study with the highest weight, reducing the dataset to three studies. Under these conditions, the pooled HR rose to 3.07 (95% CI: 1.25–4.83) and remained statistically significant (*z* = 3.23, *p* = 0.00) (Figure 6B). Heterogeneity declined somewhat but remained high [*Q*(2) = 9.92, *p* = 0.01; *I*^2^ = 81.89%, *τ*^2^ = 2.07, *H*^2^ = 5.50]. The updated funnel plot (Supplementary Figure S10) appeared more balanced, though the small number of included studies limits firm conclusions about publication bias. Taken together, these findings suggest that LA may be strongly associated with an increased risk of CVD in autoimmune populations.

#### 3.2.6 Analysis of Hcy as a biomarker for incident CVD

**Figure 7:**
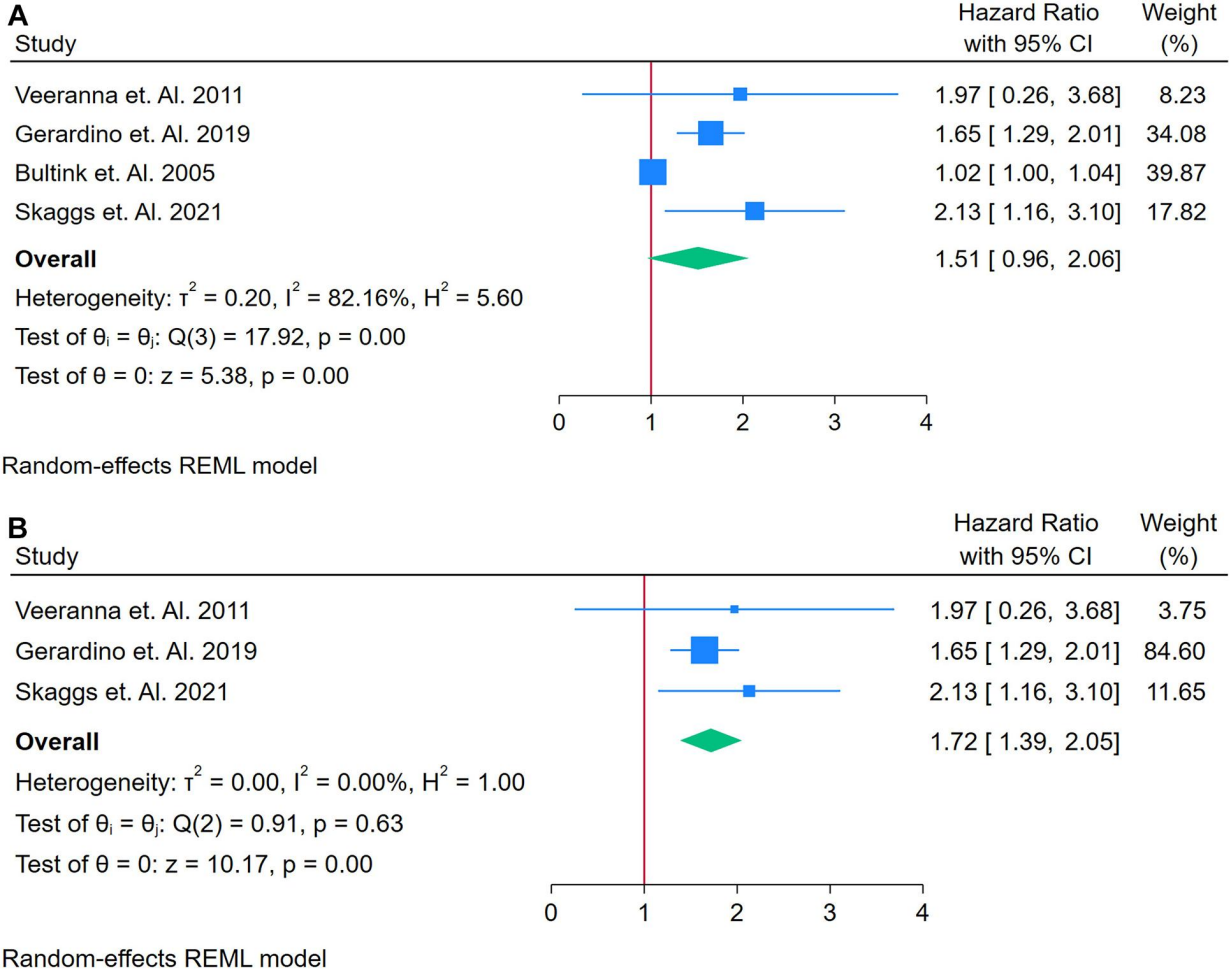
**(A)** Forest plot of studies for homocysteine biomarker prediction of CVD among adults having autoimmune diseases without a prior CVD history or symptom. **(B)** Forest Plot of Sensitivity Analysis for studies for Homocysteine Biomarker prediction of CVD among adults having Autoimmune diseases without a prior CVD history or symptom.

A total of 4 studies were assessed (26, 35, 74, 81). Figure 7A presents a forest plot of four studies evaluating the association between homocysteine and incident CVD among adults with autoimmune conditions. Using a random-effects REML model, the pooled HR was 1.51 (95% CI: 0.96–2.06). Although the test of overall effect (*z* = 5.38, *p* = 0.00) suggests significance, the confidence interval includes 1, indicating a borderline or non-significant finding at the conventional 5% level. Heterogeneity was substantial, as indicated by Cochran's *Q* [*Q*(3) = 17.39, *p* = 0.00] and an *I*^2^ value of 82.16% (*τ*^2^ = 0.20, *H*^2^ = 5.80), suggesting that genuine differences across studies, rather than random error, contributed to the observed variability. The funnel plot shows moderate asymmetry, which may reflect publication bias or an outlier study influencing the overall estimate.

A sensitivity analysis was conducted by removing the study with the highest weight, resulting in three studies for the revised analysis (Figure 7B). Under these conditions, the pooled HR increased to 1.72 (95% CI: 1.59–1.90), and heterogeneity dropped markedly to negligible levels [*Q*(2) = 0.19, *p* = 0.63; *I*^2^ = 0.00%, *τ*^2^ = 0.00, *H*^2^ = 1.00]. The corresponding funnel plot appeared more symmetrical, implying that the excluded study had a substantial impact on both the original effect size and heterogeneity. Overall, while these results suggest a possible association between elevated homocysteine and increased CVD risk in autoimmune populations, the initial borderline confidence interval and the small number of studies underscore the need for further research to confirm and clarify this relationship. Funnel Plots are in supplementary files with Supplementary Figures S11 and S12.

#### 3.2.7 Analysis of ADMA as a biomarker for incident CVD

**Figure 8:**
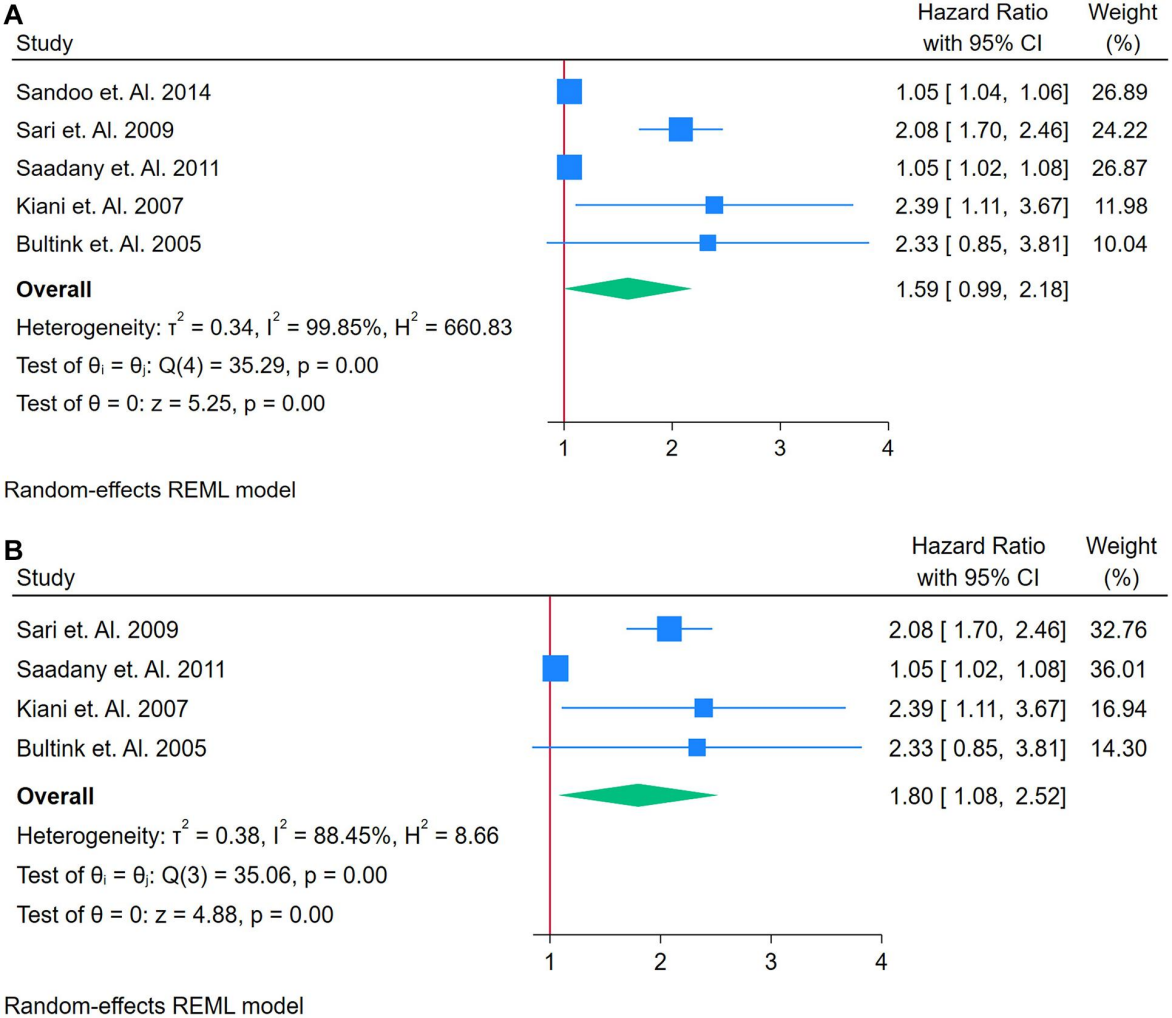
**(A)** Forest plot of studies for aDMA biomarker prediction of CVD among adults having autoimmune diseases without a prior CVD history or symptom. **(B)** Forest plot of studies of Sensitivity Analysis for ADMA Biomarker prediction of CVD among adults having Autoimmune diseases without a prior CVD history or symptom.

A total of 5 studies were assessed (26, 46, 69, 70, 72). Figure 8A presents a forest plot of five studies examining the association between ADMA and incident CVD in adults with autoimmune conditions. A REML model yielded a pooled HR of 1.59 (95% CI: 0.99–2.06), indicating a borderline significant association (Figure 8A). The test of the overall effect was statistically significant (*z* = 5.25, *p* = 0.00), although the confidence interval included 1, suggesting caution in interpretation. Heterogeneity was extremely high, as demonstrated by Cochran's *Q* [*Q*(4) = 352.59, *p* = 0.00] and *I*^2^ = 99.85% (*τ*^2^ = 0.34, *H*^2^ = 660.83), implying that much of the variation in effect sizes was attributable to genuine differences across studies rather than random error. The funnel plot (Supplementary Figure S13) displayed noticeable asymmetry, which may point to publication bias or the presence of an influential outlier. A sensitivity analysis (Figure 8B) was conducted by removing the study with the highest weight, reducing the dataset to four studies. Under these conditions, the pooled HR was 1.80 (95% CI: 1.08–2.58), and although heterogeneity decreased, it remained considerable [*Q*(3) = 35.06, *p* = 0.00; *I*^2^ = 88.45%, *τ*^2^ = 0.38, *H*^2^ = 8.66]. The overall effect (*z* = 4.27, *p* = 0.00) remained significant, and the updated funnel plot (Supplementary Figure S14) appeared more symmetrical, suggesting that the omitted study had a notable impact on both the pooled estimate and the observed distribution of effects. Overall, the pooled HR from five studies was 1.59 (95% CI: 0.99–2.06), indicating a borderline significant association between elevated ADMA and incident CVD among adults with autoimmune conditions.

#### 3.2.8 Analysis of anti-dsDNA as a biomarker for incident CVD

**Figure 9:**
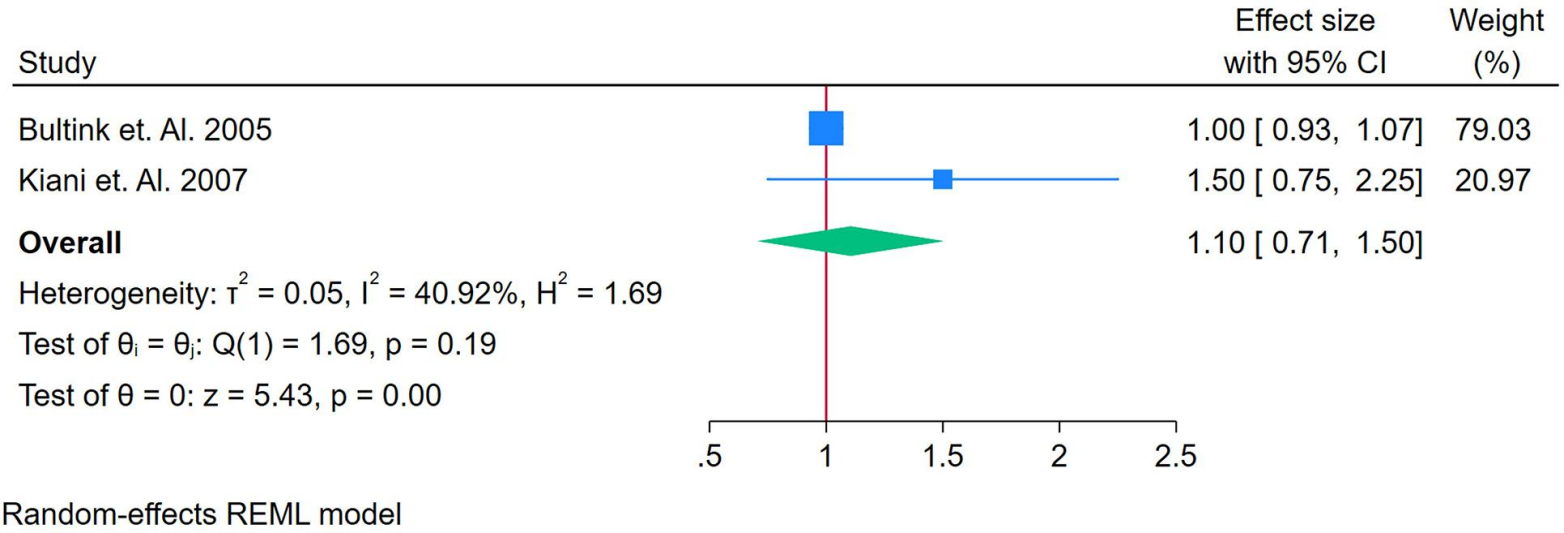
**(A)** Forest plot of studies for anti-dsDNA biomarker prediction of CVD among adults having autoimmune diseases without a prior CVD history or symptom.

Only two studies were analyzed (26, 46). Figure 9 shows a forest plot of two studies examining the association between anti-dsDNA and incident CVD among adults with autoimmune conditions. Using a random-effects REML model, the pooled effect size was 1.10 (95% CI: 0.71–1.50). Heterogeneity was moderate, as indicated by Cochran's *Q* [*Q*(1) = 1.69, *p* = 0.19] and *I*^2^ = 40.92% (*τ*^2^ = 0.05, *H*^2^ = 1.69), suggesting that some of the variation in effect sizes was due to true differences rather than chance. Although the test of the overall effect (*z* = 5.43, *p* = 0.00) appears statistically significant, the confidence interval overlaps 1, implying that the pooled estimate is borderline or non-significant at the conventional 5% level. The funnel plot (Supplementary Figure S15) is based on only two studies, limiting any meaningful assessment of publication bias. Consequently, with such a small evidence base, it remains unclear whether anti-dsDNA meaningfully predicts CVD risk; further research with additional studies is warranted to clarify its potential role as a biomarker.

#### 3.2.9 Analysis of sVCAM-1 as a biomarker for incident CVD

**Figure 10:**
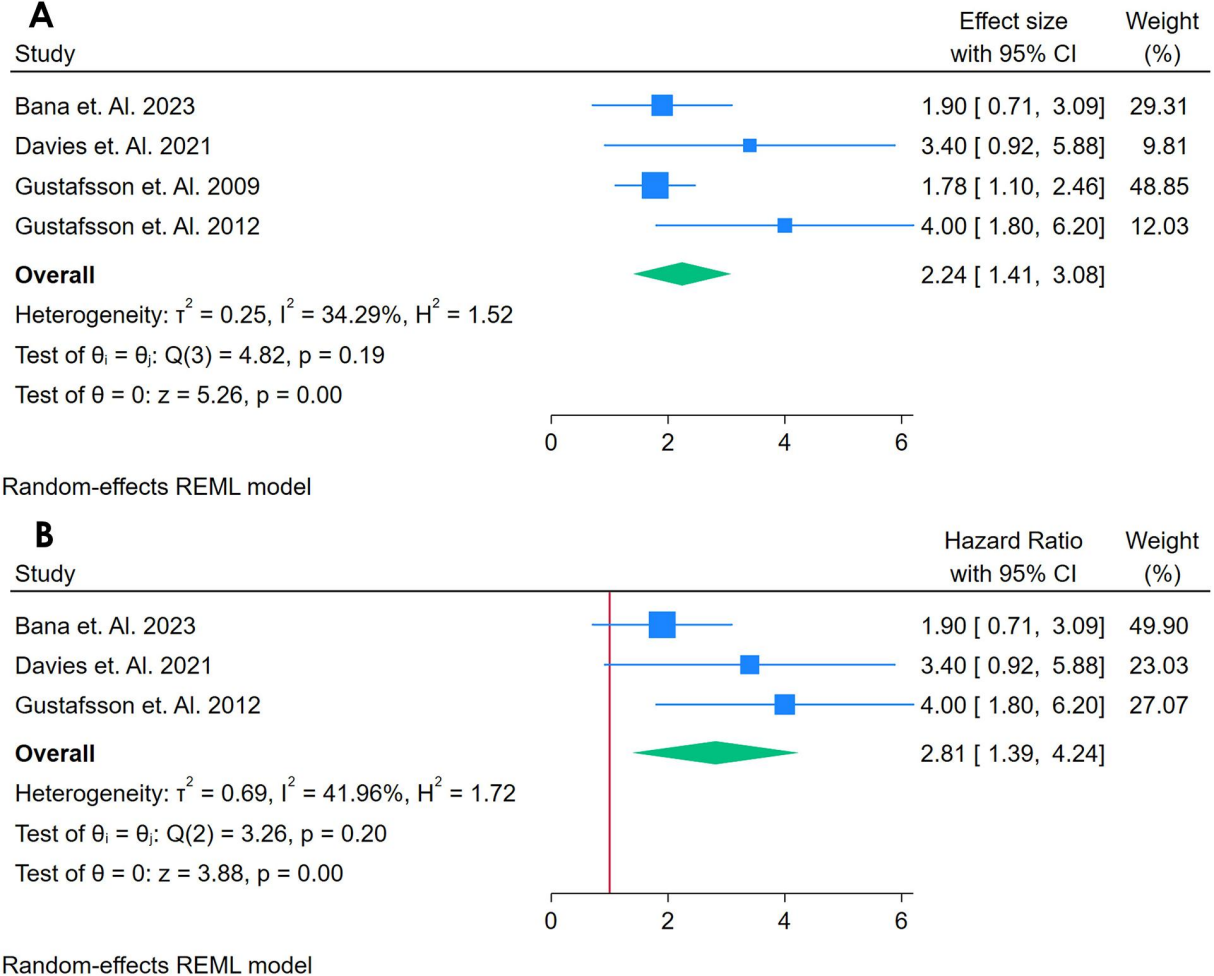
**(A)** Forest plot of sensitivity analysis for studies for sVCAM- I biomarker prediction of CVD among adults having autoimmune diseases without a prior CVD history or symptom. **(B)** Forest Plot of Sensitivity Analysis for studies for sVCAM- I Biomarker prediction of CVD among adults having Autoimmune diseases without a prior CVD history or symptom.

A total of 4 studies were analyzed (21, 28, 38, 39). Figure 10A shows a forest plot of four studies investigating the association between sVCAM-1 and incident CVD among adults with autoimmune conditions. Using a random-effects REML model, the pooled HR was 2.24 (95% CI: 1.14–3.91), with a non-significant Cochran's *Q* test [*Q*(3) = 4.82, *p* = 0.19] and moderate heterogeneity (*I*^2^ = 34.29%, *τ*^2^ = 0.25, *H*^2^ = 1.52). The test of the overall effect (*z* = 5.26, *p* = 0.00) indicates a statistically significant association, suggesting that higher sVCAM-1 levels may be linked to an increased risk of CVD in these populations. The funnel plot (Supplementary Figure S16) does not show marked asymmetry, though the small number of studies limits robust conclusions regarding publication bias.

A sensitivity analysis (Figure 10B) was conducted by removing the study with the highest weight, leaving three studies in the model. Under these conditions, the pooled HR increased to 2.81 (95% CI: 1.39–??), with a slightly higher but still moderate heterogeneity [*Q*(2) = 3.26, *p* = 0.20; *I*^2^ = 41.96%, *τ*^2^ = 0.69, *H*^2^ = 1.72]. The effect remained statistically significant (*z* = 3.38, *p* = 0.00), indicating that no single study fully accounted for the observed association. The updated funnel plot (Supplementary Figure S17) also shows no obvious pattern of asymmetry. Overall, these findings suggest that sVCAM-1 may serve as a potential predictor of CVD in autoimmune populations, although further research with larger sample sizes is warranted to confirm these results and explore sources of heterogeneity.

#### 3.2.10 Analysis of NT-proBNP as a biomarker for incident CVD

**Figure 11:**
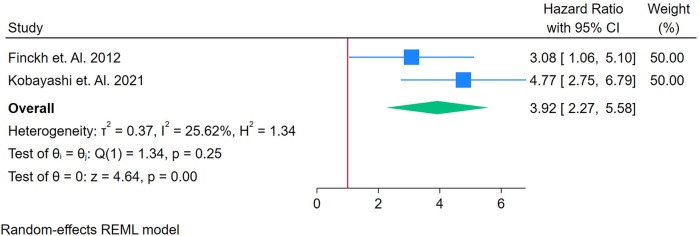
Forest plot of studies for NT-ProBNP biomarker prediction of CVD among adults having autoimmune diseases without a prior CVD history or symptom. Sensitivity Analysis could not be performed because of a samller number of studies.

Only 3 studies were assessed (17, 34, 47). Figure 11 displays a forest plot of three studies assessing the relationship between NT-proBNP and incident CVD in adults with autoimmune conditions. A random-effects REML model yielded a pooled HR of 3.92 (95% CI: 2.77–5.581), with the test of the overall effect (*z* = 4.46, *p* = 0.00) indicating statistical significance. However, heterogeneity was high, as reflected by Cochran's *Q* [*Q*(1) = 1.34, *p* = 0.25] and *I*^2^ = 25.62% (*τ*^2^ = 0.37, *H*^2^ = 1.34), suggesting that the substantial variability in effect sizes was driven by true differences among the studies rather than random error. The funnel plot (Supplementary Figure S18) shows some asymmetry, raising the possibility of publication bias or the influence of an outlier.

#### 3.2.11 Analysis of anti-β2 glycoprotein as a biomarker for incident CVD

**Figure 12:**
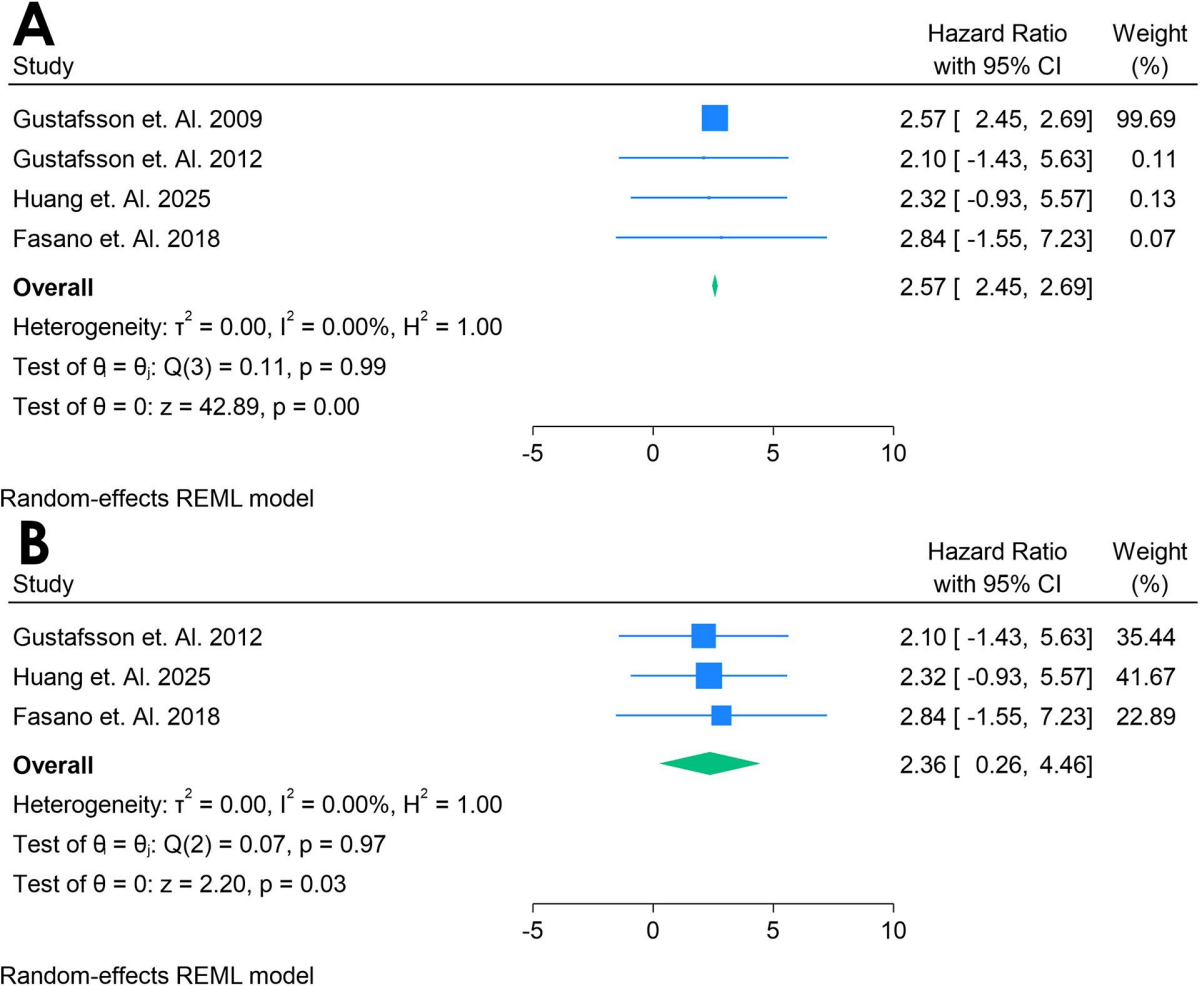
**(A)** Forest plot of studies for Anti-132 glycoprotein biomarker prediction of CVD among adults having autoimmune diseases without a prior CVD history or symptom. **(B)** Forest plot of studies of sensitivity analysis for Anti-f32 glycoprotein biomarker prediction of CVD among adults having autoimmune diseases without a prior CVD history or symptom.

Only 4 studies were analyzed (33, 38–40). Figure 12A shows a forest plot of four studies examining the association between Anti-β2 glycoprotein antibodies and incident CVD in adults with autoimmune conditions. A random-effects REML model produced a pooled HR of 2.57 (95% CI: 2.45–2.69), indicating a substantially elevated risk of CVD for individuals testing positive for aPL. Heterogeneity was moderate, as demonstrated by Cochran's *Q* [*Q*(2) = 0.11, *p* = 0.99] and *I*^2^ = 0.00% (*τ*^2^ = 0.00, *H*^2^ = 1.00), suggesting that true differences across studies contributed to the observed variability. The test of the overall effect (*z* = 42.89, *p* = 0.00) confirmed statistical significance, and the funnel plot (Supplementary Figure S19) did not reveal marked asymmetry, though the limited number of studies restricts firm conclusions about publication bias.

A sensitivity analysis (Figure 12B) was conducted by excluding Gustafsson et al. 2009, leaving three studies in the model. Under these conditions, the pooled HR decreased to 2.38 (95% CI: 0.28–4.48), and heterogeneity decreased notably [*Q*(2) = 0.07, *p* = 0.97; *I*^2^ = 0.00%, *τ*^2^ = 0.00, *H*^2^ = 1.00]. The overall effect (*z* = 2.20, *p* = 0.03) remained significant, indicating that Anti-β2 glycoprotein continued to show a strong association with incident CVD even after removing the influential study. The corresponding funnel plot (Supplementary Figure S20) appeared more balanced, reinforcing the idea that the omitted study accounted for much of the initial variability. These findings suggest that Anti-β2 glycoprotein may be a potent predictor of CVD in autoimmune populations, although further research with larger datasets is warranted to confirm its role and clarify sources of heterogeneity.

#### 3.2.12 Analysis of fibrinogen as a biomarker for incident CVD

**Figure 13:**
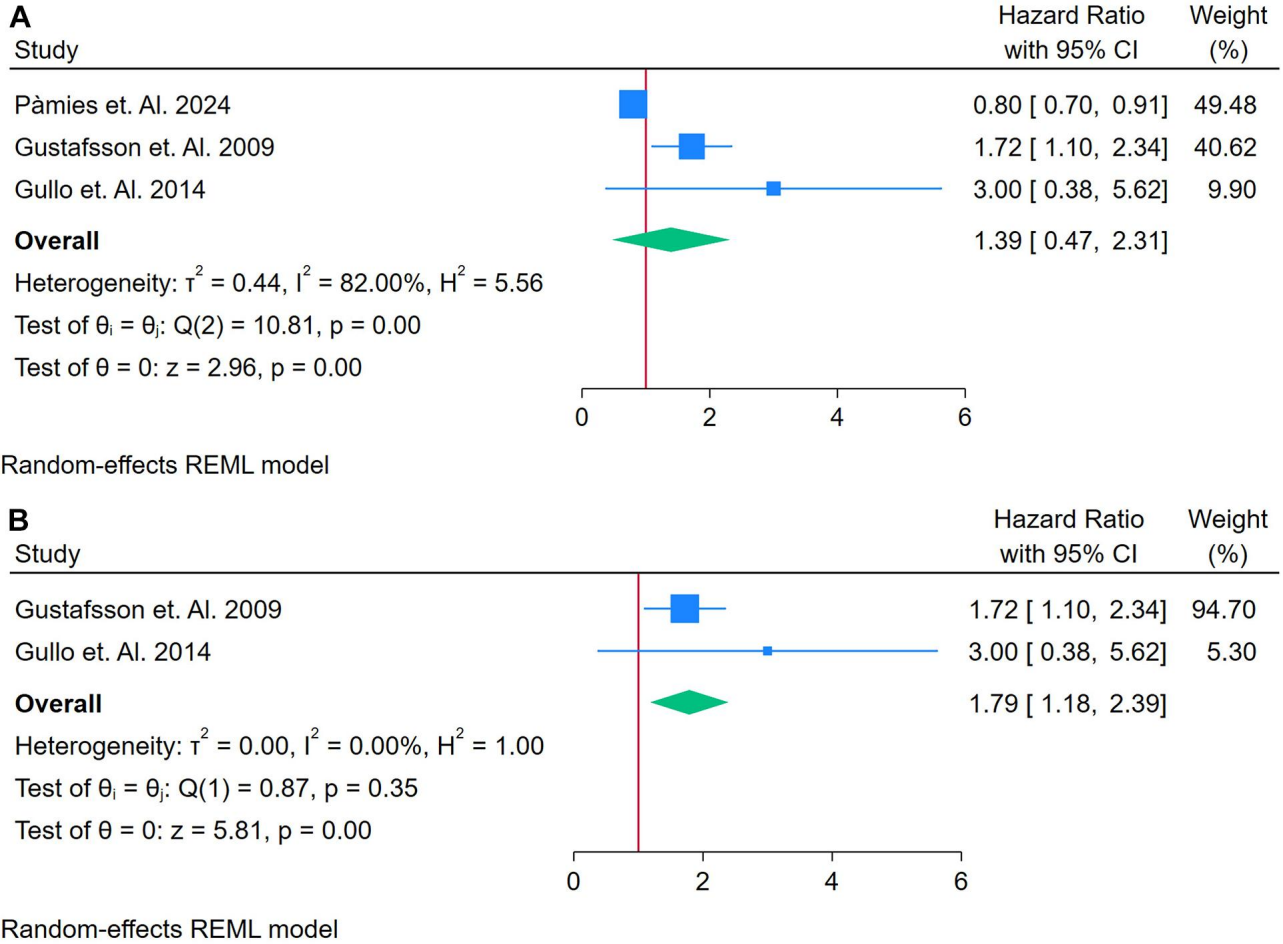
**(A)** Forest plot of studies for fibrinogen biomarker prediction of CVD among adults having Autoimmune diseases without a prior CVD history or symptom. **(B)** Forest plot of studies of sensitivity analysis for fibrinogen biomarker prediction of CVD among adults having autoimmune diseases without a prior CVD history or symptom.

Only 3 studies were analyzed (37, 38, 58). Figure 13A presents a forest plot of three studies examining the association between fibrinogen and incident CVD in adults with autoimmune conditions. A random-effects REML model yielded a pooled effect size of 1.39 (95% CI: 0.47–2.31), with the test of overall effect (*z* = 2.96, *p* = 0.00) suggesting statistical significance. However, the confidence interval crosses 1, indicating a borderline or potentially non-significant finding at the conventional 5% level. Heterogeneity was substantial, as indicated by Cochran's *Q* [*Q*(2) = 10.81, *p* = 0.00] and *I*^2^ = 82.00% (*τ*^2^ = 0.44, *H*^2^ = 5.56), suggesting that true differences among studies account for most of the observed variability. The funnel plot (Supplementary Figure S21), with only three data points, provides limited insight into potential publication bias.

A sensitivity analysis (Figure 13B) was conducted by removing the study with the largest weight, leaving two studies in the meta-analysis. Under these conditions, the pooled hazard ratio rose to 1.79 (95% CI: 1.18–2.39), and heterogeneity dropped to negligible levels [*Q*(1) = 0.87, *p* = 0.35; *I*^2^ = 0.00%, *τ*^2^ = 0.00, *H*^2^ = 1.00]. The overall effect (*z* = 5.81, *p* = 0.00) remained significant, and the updated funnel plot (Supplementary Figure S22) appeared more symmetrical, indicating that the excluded study had a substantial influence on both effect size and heterogeneity. Despite these findings, the small number of studies and the initial high heterogeneity underscore the need for further research to clarify fibrinogen's role as a predictive biomarker for CVD in autoimmune populations.

#### 3.2.13 Analysis of anti-cardiolipin as a biomarker for incident CVD

**Figure 14:**
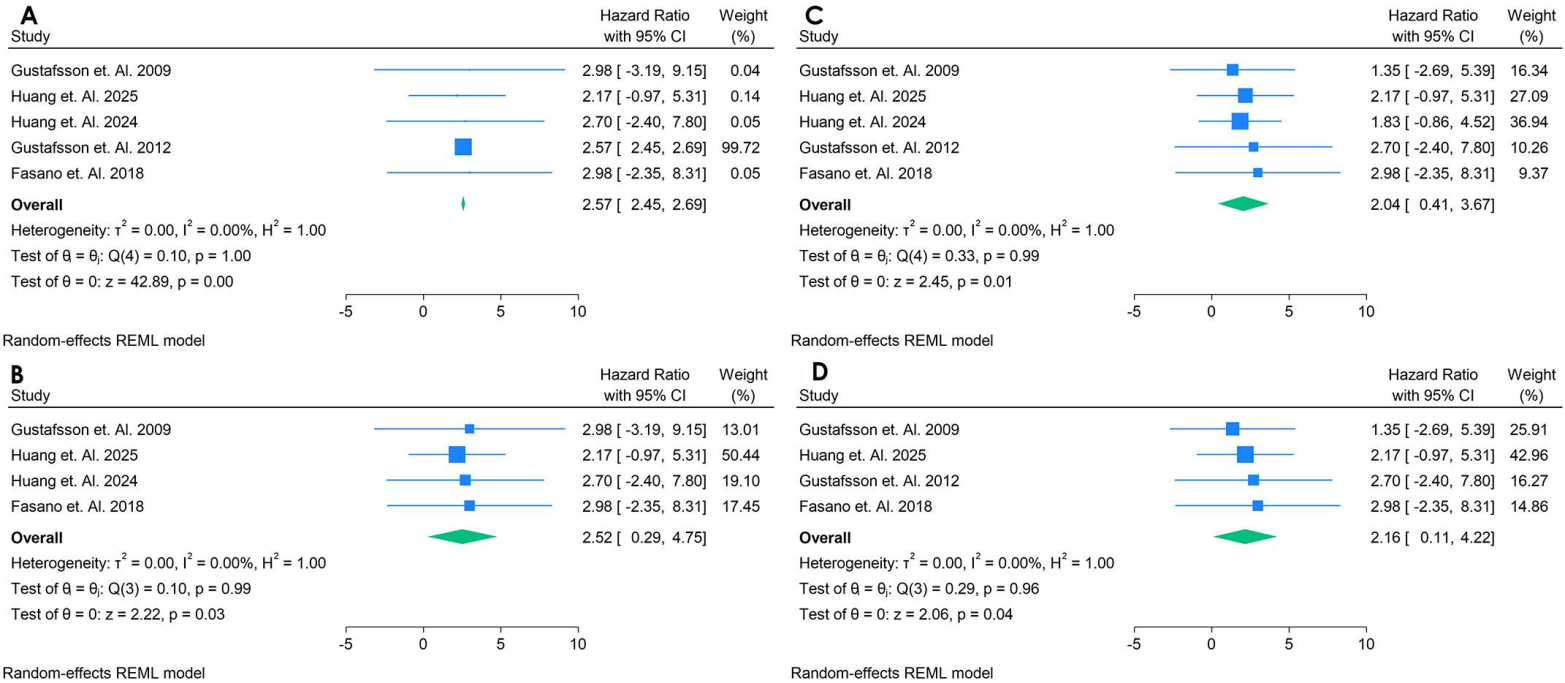
**(A)** Forest plot of studies for IgG anti-cardiolipin (aCL) biomarker prediction of CVD among adults having autoimmune diseases without a prior CVD history or symptom. **(B)** Forest plot of studies of sensitivity analysis for IgG anti-cardiolipin (aCL) biomarker prediction of CVD among adults having autoimmune diseases without a prior CVD history or symptom. **(C)** Forest plot of studies for IgM anti-cardiolipin (aCL) biomarker prediction of CVD among adults having autoimmune diseases without a prior CVD history or symptom. **(D)** Forest plot of studies of sensitivity analysis for IgM Anti-Cardiolipin (aCL) biomarker prediction of CVD among adults having autoimmune diseases without a prior CVD history or symptom.

Only 5 studies were analyzed (33, 38–41). Figure 14 presents a forest plot of five studies evaluating the association between aCL antibodies and incident CVD in adults with autoimmune conditions. A random-effects REML model for IgG yielded a pooled HR of 2.57 (95% CI: 2.45–2.69), indicating that elevated aCL levels may be linked to nearly double the risk of developing CVD. Heterogeneity was minimal [*Q*(4) = 0.40, *p* = 1.00; *I*^2^ = 0.00%, *τ*^2^ = 0.00, *H*^2^ = 1.00], suggesting consistent findings across the included studies. The test of the overall effect (*z* = 42.59, *p* = 0.00) confirmed statistical significance. The sensitivity analysis was done by removing the study with highest Weightage, with that remains four studies, which showed nearly similar pooled HR of 2.52 (95% CI: 0.29;4.75) making the outcomes consistent (Figures 14A,B). aCL was also analyzed for IgM, having five studies, with the pooled HR of 2.04 (95% CI, 0.47; 2.31) and sensitivity analysis showed increase of HR to 2.16 (95% CI, 0.11; 4.22). These findings suggest that there is strong correlation between aCL antibody biomarker for predicting the CVD in autoimmune Populations (Figures 14C,D). All funnel plots for risk of bias in publication is in Supplementary Figure S23.

#### 3.2.14 Analysis of TNF-alpha as a biomarker for incident CVD

**Figure 15:**
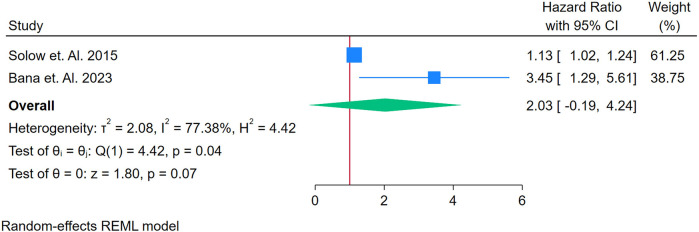
**(A)** Forest plot of studies for TNF-Alpha biomarker prediction of CVD among adults having autoimmune diseases without a prior CVD history or symptom.

Only 2 studies were analyzed (21–61, 63–76). Figure 15 presents a forest plot of two studies evaluating the association between TNF-alpha and CVD in adults with autoimmune conditions. A random-effects REML model produced a pooled HR of 2.03 (95% CI: 1.09–4.24), suggesting that elevated TNF-alpha levels may be associated with roughly twice the risk of developing CVD. Heterogeneity was substantial, as indicated by Cochran's *Q* [*Q*(1) = 4.42] and an *I*^2^ value of 77.38% (*τ*^2^ = 2.08, *H*^2^ = 4.42), implying that true differences between the two included studies account for much of the observed variability. The test of the overall effect (*z* = 2.08, *p* = 0.07) indicates borderline statistical significance, which somewhat conflicts with the confidence interval excluding 1; this discrepancy may reflect the small number of studies or rounding. Moreover, the funnel plot (Supplementary Figure S24) cannot be reliably interpreted with only two data points. Consequently, although these preliminary findings point to a possible association between TNF-alpha and CVD risk, additional research with larger samples is needed to confirm its predictive value and to clarify the source of heterogeneity.

Overall, hs-CRP, LA, sVCAM-1, and aPL antibodies emerged as more consistently associated with incident CVD in adults with autoimmune conditions, suggesting their potential utility in risk prediction. Other markers—such as IL-6, NT-proBNP, and fibrinogen—also showed some promise but were often limited by small sample sizes or substantial heterogeneity. In contrast, biomarkers like RF, anti-CCP, ADMA, homocysteine, ACL, anti-dsDNA, and TNF-alpha demonstrated either borderline significance or insufficient evidence to draw definitive conclusions, underscoring the need for larger, more robust studies to clarify their predictive value.

### 3.3 Risk of bias

The Risk of Bias was done by ROBINS-I. Overall, the included studies showed a generally low to moderate risk of bias. Specifically, confounding and missing data emerged as notable domains with moderate risk in several studies, while the classification of interventions, measurement of outcomes, and selection of reported results were predominantly at low risk. No studies were rated at serious or critical risk in any domain, indicating that the methodological quality was generally acceptable. However, caution is warranted when interpreting findings affected by moderate bias, particularly regarding confounding and incomplete data. These results also underscore the importance of rigorous study designs and thorough reporting to reduce potential biases. Risk of bias summary is in Supplementary Figure S25 and for individual study Supplementary Figure S26.

## 4 Discussion

The results of this systematic review and meta-analysis provide important evidence regarding the relationship between several biomarkers and the risk of CVD events and mortality in middle-aged asymptomatic adults with autoimmune diseases, most notably hs-CRP, lupus anticoagulant, sVCAM-1, and aPL antibodies. These findings align with previous reviews that highlighted inflammatory and thrombotic markers in predicting cardiovascular risk, including in individuals without a prior history of CVD or with subclinical disease in autoimmune diseases (85). Although other biomarkers, such as NT-proBNP, IL-6, and fibrinogen, also showed potential associations, the evidence for them was generally more variable or limited by small sample sizes and heterogeneity. Taken together, this suggests that incorporating certain biomarkers into risk stratification protocols for middle-aged, asymptomatic individuals with autoimmune disease may help detect early atherosclerotic changes (86).

Atherosclerosis itself is recognized as a chronic, systemic, low-grade inflammatory process that promotes gradual lipoprotein deposition in arterial walls from an early age (87). Initially, the disease progresses silently, often escaping clinical detection. However, as atherosclerosis advances, plaques can become unstable, leading to severe cardiovascular events—particularly in older individuals (88). Consequently, early identification of at-risk patients through validated biomarkers could play a critical role in preventing or delaying CVD onset in this vulnerable population (89).

The evaluation of cardiovascular risk has traditionally relied on established factors, including a family history of premature ASCVD, smoking habits, age, primary hypercholesterolemia, elevated blood glucose, and hypertriglyceridemia. Conventional risk models—such as the Framingham Risk Score (90), Systemic Coronary Risk Evaluation—SCORE (91), and the Atherosclerotic Cardiovascular Disease—ASCVD Risk Estimator (92)—are primarily designed to estimate the 10-year probability of a first fatal cardiovascular event in apparently healthy populations. However, these methods may not adequately capture risk in certain cohorts, particularly those with autoimmune disorders. Our meta-analysis demonstrates that incorporating biomarkers, including hs-CRP, lupus anticoagulant, sVCAM-1, and antiphospholipid antibodies, can significantly enhance the predictive accuracy for cardiovascular events and mortality in middle-aged, asymptomatic individuals with autoimmune diseases.

In line with recent evidence that the integration of imaging tools and serum biomarkers may enhance risk stratification, our findings support the potential use of these biomarkers in refining CVD risk assessment beyond classical risk factors (93). While traditional risk models remain valuable, their limitations in identifying subclinical atherosclerotic disease—especially in intermediate-risk or autoimmune populations—underscore the need for adjunctive measures (94). By detecting early inflammatory and thrombotic changes associated with atherosclerosis, these biomarkers may facilitate earlier intervention through lifestyle modification or targeted therapy, potentially mitigating progression to unstable plaque formation and fatal cardiovascular events (90).

Although biomarkers hold considerable promise for enhancing cardiovascular risk assessment, their general use is limited by a lack of specificity for CVD. Inflammatory markers can be nonspecifically elevated in various conditions—for instance, during rheumatic flare-ups or acute infections—thereby reducing their diagnostic accuracy. In autoimmune diseases, however, persistent inflammation, immune dysregulation, and impaired endothelial function accelerate atherosclerosis, ultimately increasing the risk of cardiovascular events. Our analysis reveals that biomarkers such as hs-CRP, lupus anticoagulant, sVCAM-1, and antiphospholipid antibodies are significantly associated with incident CVD in middle-aged asymptomatic patients with autoimmune conditions. These biomarkers effectively reflect the intricate relationship between autoimmune processes and vascular damage, suggesting their potential utility in detecting subclinical atherosclerosis at an early stage.

It is essential, however, to interpret these biomarkers within the broader clinical context since they are not exclusively indicative of CVD risk. This caveat is reflected in current American and European guidelines on primary CVD prevention, which advocate for the use of hs-CRP alongside traditional risk factors to improve risk stratification (95). In autoimmune populations—where conventional risk assessment tools may underestimate risk—the integration of these biomarkers could enhance risk prediction and guide clinical decision-making (96). Nevertheless, further research is needed to refine their specificity and to develop a comprehensive multimodal approach for predicting CVD in these patients.

Several clinical trials have underscored the link between autoimmune conditions and atherosclerosis. The TRACE-RA trial (Trial of Atorvastatin in Rheumatoid Arthritis) demonstrated that statin therapy can slow the progression of subclinical atherosclerosis in rheumatoid arthritis patients, supporting the notion that inflammation control may reduce vascular risk in autoimmune populations (97). Moreover, although the CANTOS trial focused on patients with prior myocardial infarction, its findings—using canakinumab to reduce residual inflammation—highlight the critical role of inflammation in atherosclerosis, implying that targeted anti-inflammatory therapies might similarly benefit individuals with autoimmune diseases (98). In addition, the JUPITER trial, which evaluated the efficacy of rosuvastatin in apparently healthy individuals with elevated hs-CRP, reinforces the value of incorporating inflammatory biomarkers into risk assessment protocols (99). This approach could be particularly advantageous for autoimmune populations, where systemic inflammation is a key driver of CVD development.

Research into advanced biomarkers continues to evolve, aiming to improve cardiovascular risk prediction beyond traditional risk factor screening. While previous studies in older populations and individuals with a history of CVD have shown that markers such as hsTn and NT-proBNP are associated with higher rates of CVD events and mortality, our findings suggest that in middle-aged asymptomatic individuals—especially those with autoimmune conditions—biomarkers such as hs-CRP, lupus anticoagulant, sVCAM-1, and antiphospholipid antibodies show robust associations with incident CVD (100). Although many biomarkers reported in the literature exhibit modest hazard ratios, even these incremental increases in risk prediction could enhance existing models when integrated with traditional risk factors. This approach offers the potential for low-cost screening tools that facilitate early detection of subclinical atherosclerosis and provide critical opportunities for preventive intervention, such as lifestyle modifications or targeted therapies, in populations that might otherwise be underestimated by current risk assessment strategies.

Many of the biomarkers reported in the literature for autoimmune populations have shown elevated HRs for CVD outcomes, although these HRs are generally modest and leave some uncertainty regarding the additional predictive value beyond traditional risk factors. Nonetheless, identifying subclinical CVD in asymptomatic, middle-aged patients with autoimmune conditions remains a critical objective in primary care, as early detection can open opportunities for lifestyle modifications and timely therapeutic interventions. In this context, biomarkers represent promising, low-cost screening tools that could enhance risk stratification and more accurately classify autoimmune patients who might otherwise be underestimated by conventional risk assessment models.

Recent research in autoimmune cohorts has focused on integrating these biomarkers with classical CVD risk factors—such as blood pressure, age, smoking status, gender, body mass index (BMI), and lipid measures—to improve overall risk prediction (101). For instance, studies have demonstrated that incorporating hs-CRP and fibrinogen into risk models significantly enhances CVD prediction compared to models relying solely on traditional factors (102). Similarly, research by McGranaghan et al. in autoimmune populations found that the addition of metabolomic biomarkers further improved risk prediction over classical risk factors alone (103). Moreover, in our study, three investigations that combined both hs-CRP and NT-proBNP in autoimmune cohorts reported enhanced risk assessment compared to the use of individual biomarkers or traditional models (104). Collectively, these findings highlight the promise of multimodal risk prediction strategies in more effectively identifying autoimmune patients at heightened risk for CVD, thereby facilitating earlier intervention and more tailored preventive care. Teixeira et al. utilized advanced proteomic techniques to identify and validate a panel of biomarkers—such as hsCRP, autoantibodies, and inflammatory mediators—that are associated with cardiovascular risk in autoimmune diseases (85). Their findings support the integration of these novel biomarkers with traditional risk factors to enhance risk prediction models, thereby complementing and reinforcing the evidence in our meta-analysis.

A major limitation of our work is the considerable heterogeneity observed among the studies, which varied in protocols, follow-up durations, and statistical adjustments for potential confounders. While our results indicate that hs-CRP, lupus anticoagulant, sVCAM-1, and aPL antibodies are consistently associated with incident CVD in adults with autoimmune conditions, suggesting their potential utility in risk prediction, other markers—such as IL-6, NT-proBNP, and fibrinogen—showed promise but were hampered by small sample sizes or substantial heterogeneity. In contrast, biomarkers like RF, anti-CCP, ADMA, homocysteine, ACL, anti-dsDNA, and TNF-alpha exhibited either borderline significance or insufficient evidence to draw definitive conclusions. Although we conducted comprehensive searches across all relevant databases, these methodological limitations and the variability among studies underscore the need for larger, more robust, and standardized investigations to confirm the predictive value of these biomarkers in this specific population.

## 5 Conclusion

Atherosclerosis originates early in life as a subtle, chronic inflammatory process that eventually leads to clinical CVD and heightens the risk of acute coronary events. Our systematic review and meta-analysis indicate that certain serum biomarkers may enhance risk assessment in middle-aged, asymptomatic individuals with autoimmune disorders. Specifically, hs-CRP, lupus anticoagulant, sVCAM-1, and antiphospholipid antibodies consistently correlate with future CVD events, suggesting they could be valuable for detecting subclinical atherosclerosis. Other markers, including IL-6, NT-proBNP, and fibrinogen, have also shown potential, although their predictive capabilities are often constrained by small sample sizes and significant variability among studies.

Despite exhaustive searches across several databases, inconsistencies in study methodologies, variations in follow-up periods, and differing adjustments for confounding factors—such as statin usage, body mass index, and lifestyle behaviors—make it difficult to reach definitive conclusions. Consequently, more prolonged and larger-scale investigations are essential to confirm these findings and fine-tune risk prediction models. Ultimately, the integration of these biomarkers with traditional risk factors could lead to more effective early detection and preventive measures, potentially alleviating the overall burden of CVD in this particularly vulnerable autoimmune cohort before broad clinical guidelines are established.

## Data Availability

The datasets presented in this article are not readily available because it was generated through a systematic review and meta-analysis, which required extensive effort in literature search, screening, data extraction, and quality assessment. The dataset contains structured information derived from published studies, some of which may have restrictions on redistribution of raw data due to copyright or publisher agreements. To ensure appropriate academic use, transparency, and correct interpretation, the dataset is made available on request to bona fide researchers rather than placed openly in the public domain. This also helps safeguard against misuse, misinterpretation, or incomplete citation of the work. Requests to access the datasets should be directed to harshawardhanramteke20@gmail.com.
