# Supplementary material for: Systematic review and meta-analysis of cardiovascular associated biomarkers in adults with asymptomatic autoimmune diseases
Source: Front Cardiovasc Med. 2025 Oct 16;12:1598590. doi: 10.3389/fcvm.2025.1598590 (PMC12571752; doi:10.3389/fcvm.2025.1598590)
Supplement: Supplementary file 1 [file Datasheet1.pdf]

Supplementary Material

1 Supplementary Data

Search String

((HCY OR homocysteine) OR (Anti-cpp OR "anti-cyclic citrullinated peptide") OR (IL-6 OR interleukin-6) OR (ADMA OR "asymmetric dimethylarginine") OR (dsDNA OR "double stranded DNA") OR (sVCAM OR "soluble vascular cell adhesion molecule") OR (NTprobnp OR NT-proBNP OR "N-terminal pro b-type natriuretic peptide") OR (apl OR "antiphospholipid antibodies" OR Anti-Cardiolipin Antibodies OR aCL) OR (tnfalpha OR "tumor necrosis factor alpha") OR fibrinogen OR (acl OR anticardiolipin) OR rf OR (hscrp OR "high sensitivity CRP") OR "lupus anticoagulant") AND ("autoimmune diseases" OR "autoimmune disorders" OR "systemic lupus erythematosus" OR "rheumatoid arthritis" OR "multiple sclerosis" OR psoriasis OR "inflammatory bowel disease" OR "systemic sclerosis" OR "Hashimoto thyroiditis" OR "Graves disease") AND ("subclinical atherosclerosis" OR "early detection" OR atherosclerosis OR "cardiovascular risk" OR "cardiovascular disease") AND ("screening"[All Fields] OR "prediction"[All Fields] OR "predict"[All Fields]) AND ("cardiovascular diseases"[All Fields] OR "cardiovascular disease"[All Fields] OR "heart disease"[All Fields] OR mortality[All Fields]) AND English[lang] AND ("adult"[MeSH Terms] OR "middle aged"[MeSH Terms]) AND hasabstract[text] AND "2000/01/01"[PDat]:"2024/11/01"[PDat] AND "humans"[MeSH Terms]

2 Supplementary Figures and Tables

2.1 Supplementary Figures

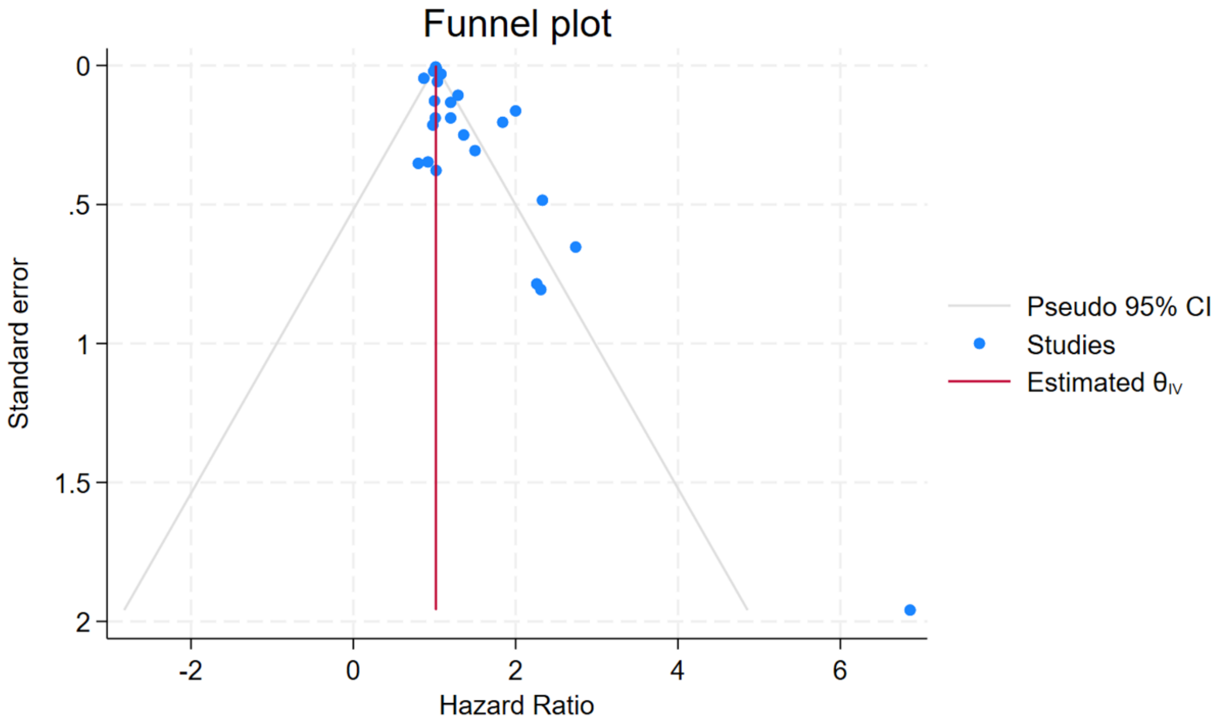

Figure S1. Funnel plot of studies for hsCRP Biomarker prediction of CVD among adults having Autoimmune diseases without a prior CVD history or symptom.

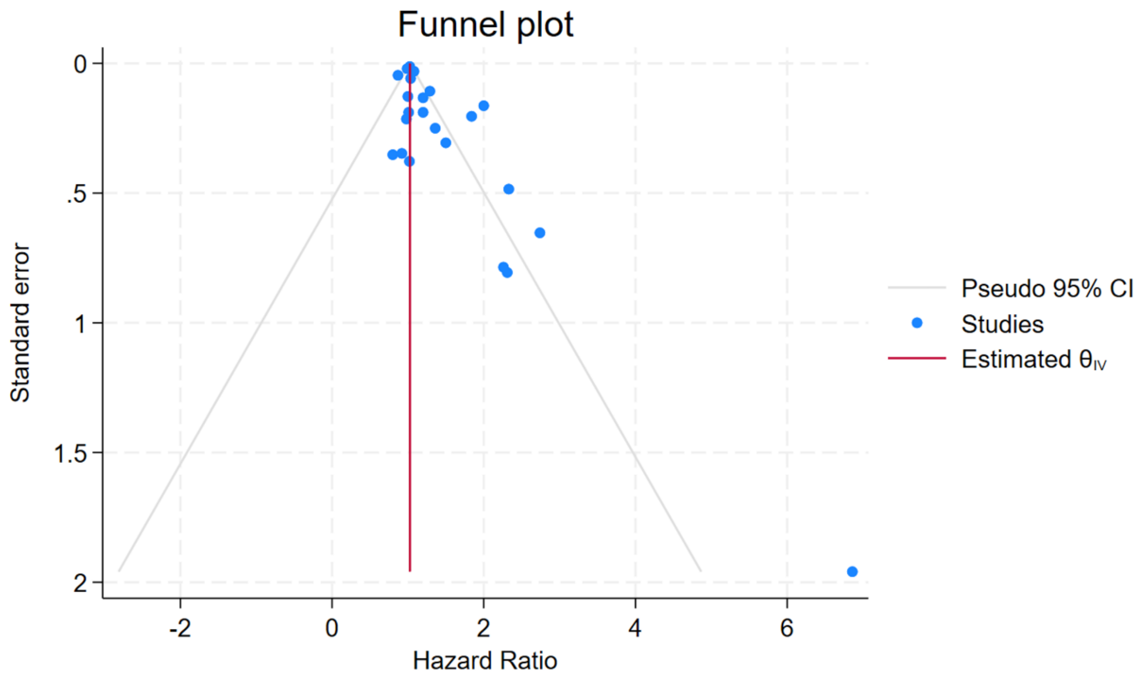

Figure S2. Funnel plot of Sensitivity Analysis of studies for hsCRP Biomarker prediction of CVD among adults having Autoimmune diseases without a prior CVD history or symptom.

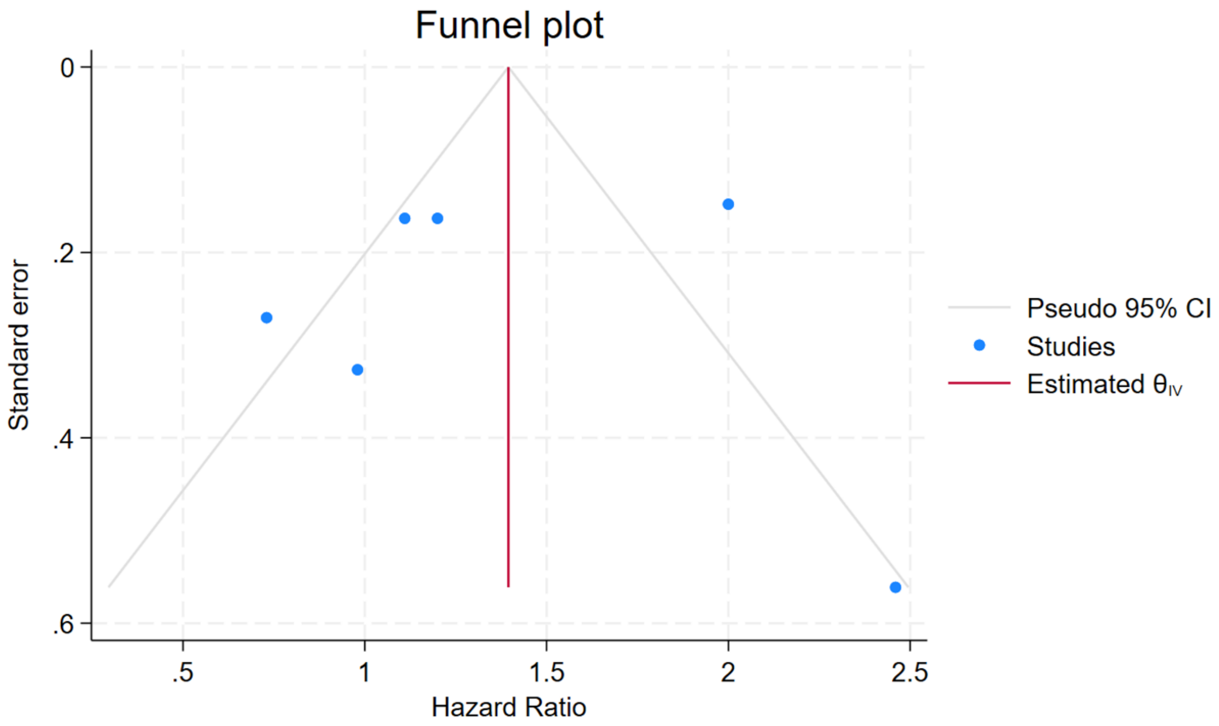

Figure S3. Funnel plot of studies for RF Biomarker prediction of CVD among adults having Autoimmune diseases without a prior CVD history or symptom.

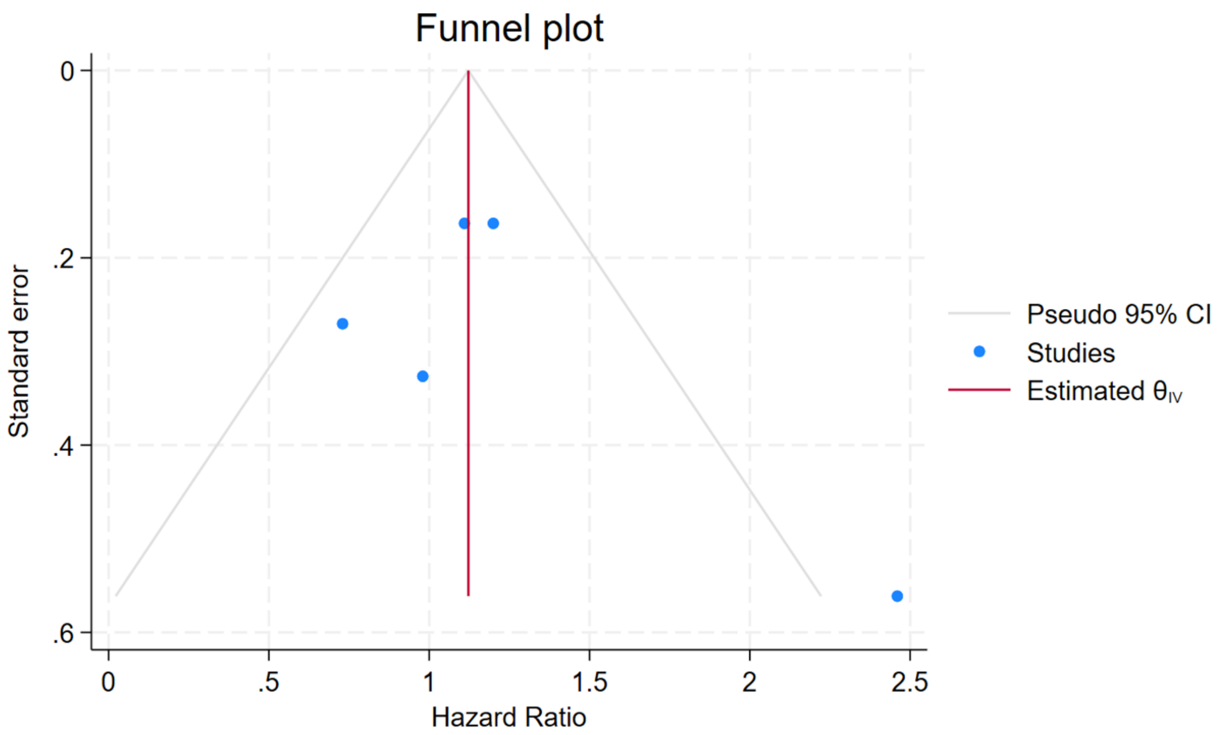

Figure S4. Funnel plot of Sensitivity Analysis of studies for RF Biomarker prediction of CVD among adults having Autoimmune diseases without a prior CVD history or symptom.

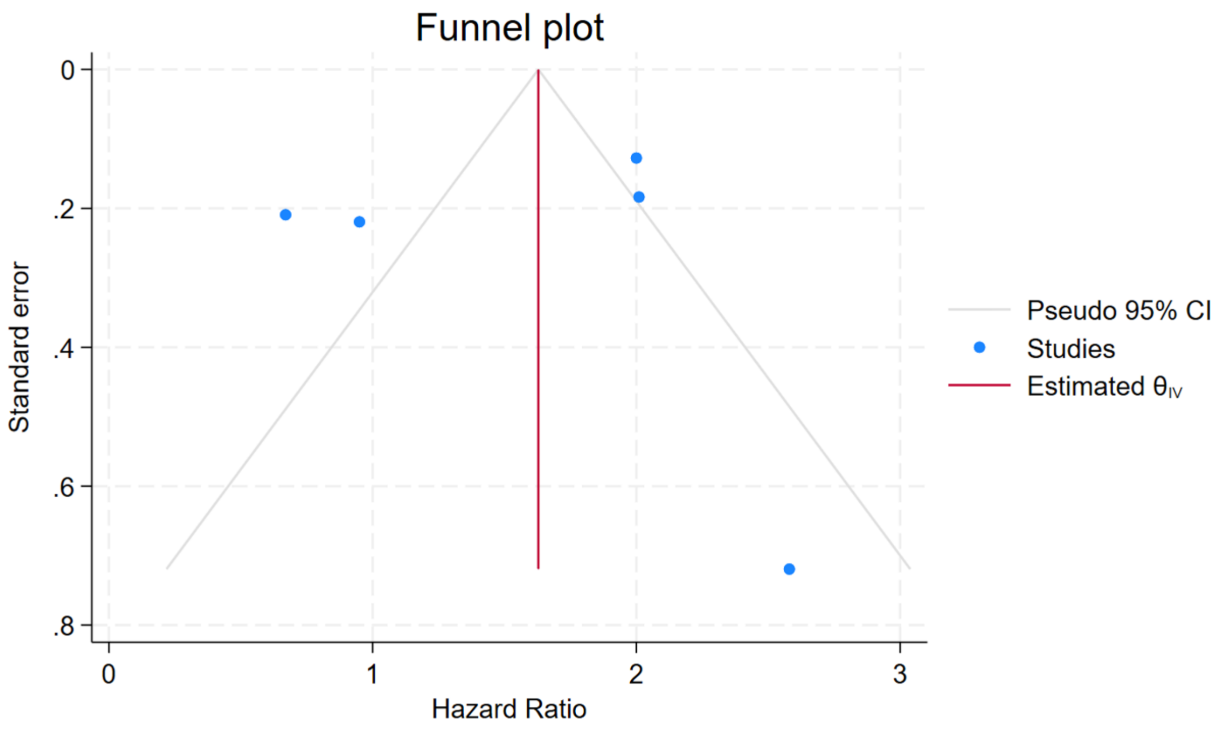

Figure S5. Funnel plot of studies for Anti-CCP Biomarker prediction of CVD among adults having Autoimmune diseases without a prior CVD history or symptom.

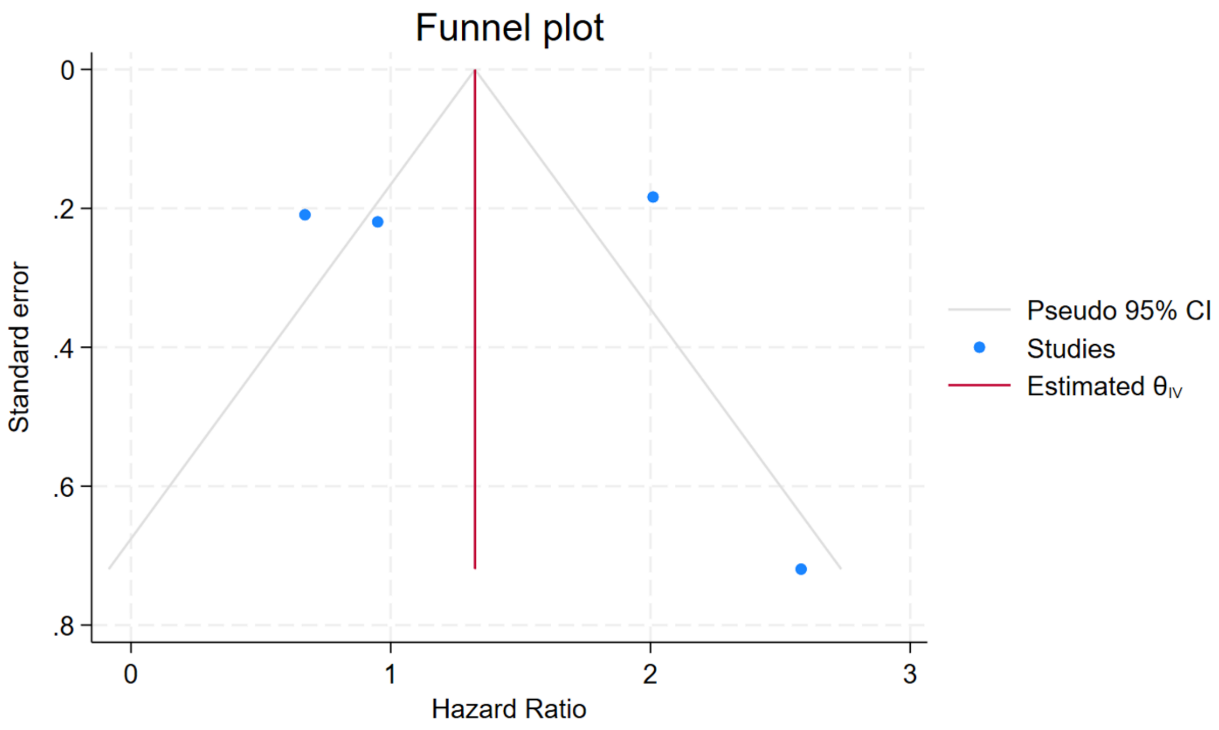

Figure S6. Funnel plot of Sensitivity Analysis of studies for Anti-CCP Biomarker prediction of CVD among adults having Autoimmune diseases without a prior CVD history or symptom.

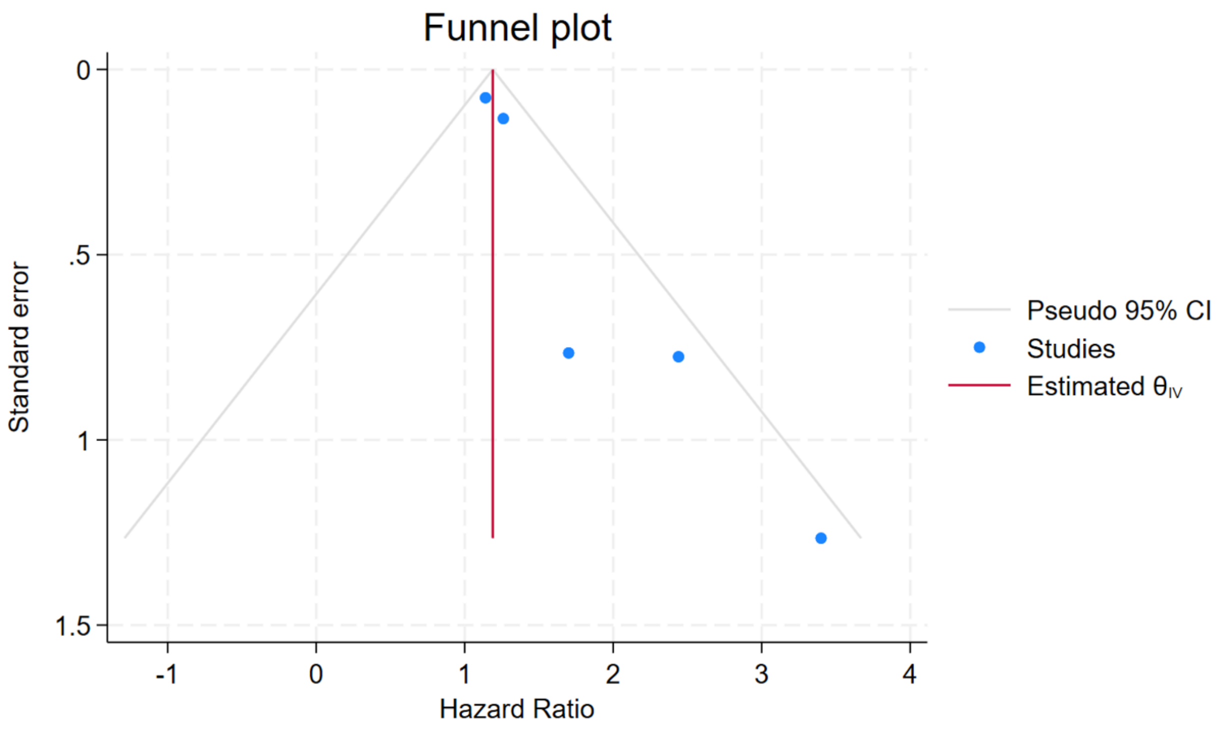

Figure S7. Funnel plot of studies for IL-6 Biomarker prediction of CVD among adults having Autoimmune diseases without a prior CVD history or symptom.

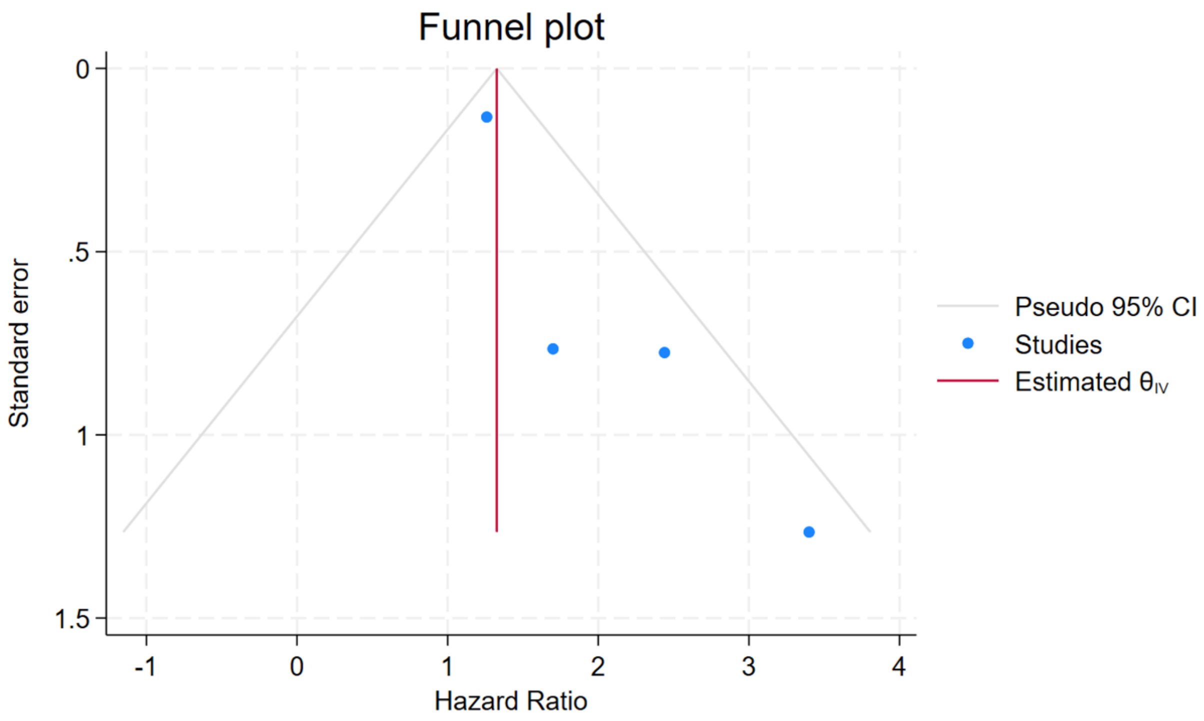

Figure S8. Funnel plot of Sensitivity Analysis of studies for IL-6 Biomarker prediction of CVD among adults having Autoimmune diseases without a prior CVD history or symptom.

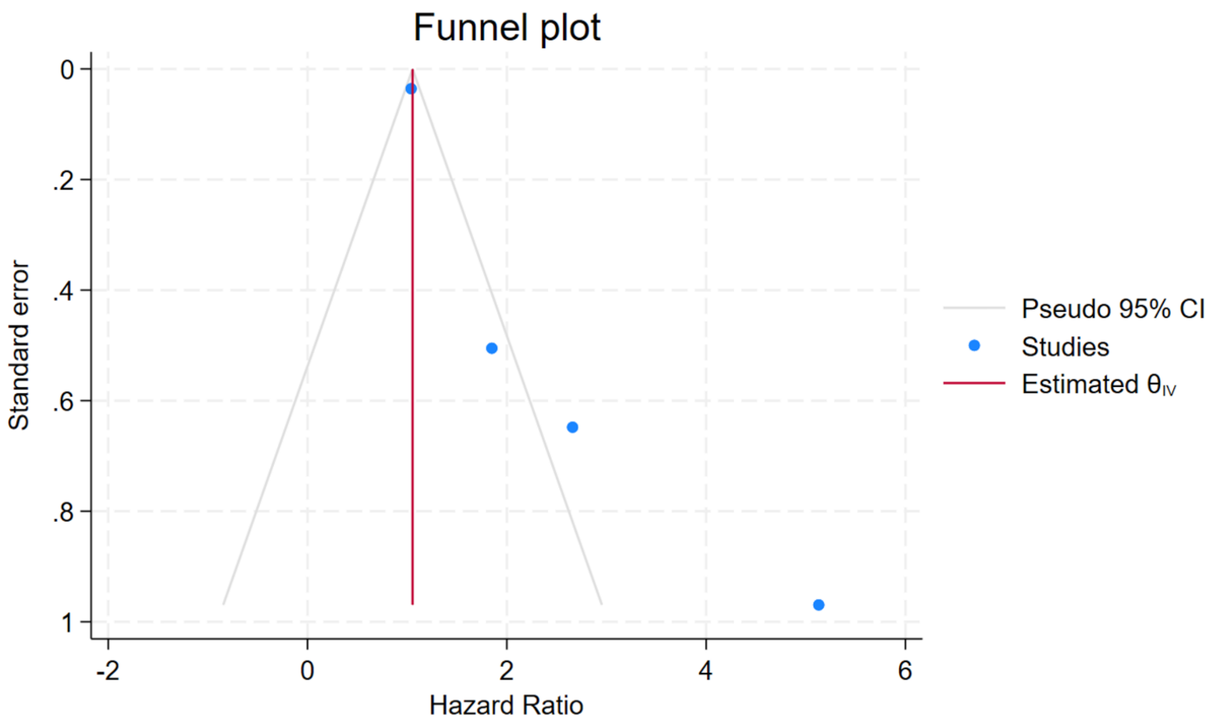

Figure S9. Funnel plot of studies for Lupus Anti-Coagulant Biomarker prediction of CVD among adults having Autoimmune diseases without a prior CVD history or symptom.

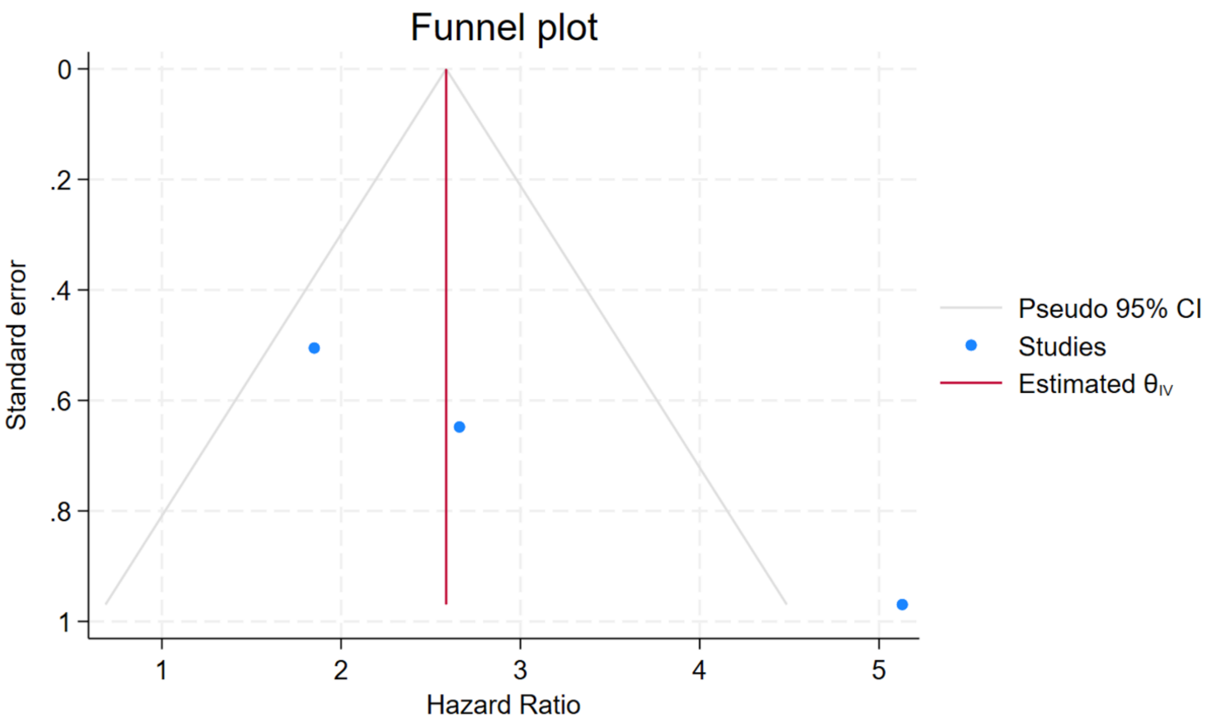

Figure S10. Funnel plot of Sensitivity Analysis of studies for Lupus Anticoagulant Biomarker prediction of CVD among adults having Autoimmune diseases without a prior CVD history or symptom.

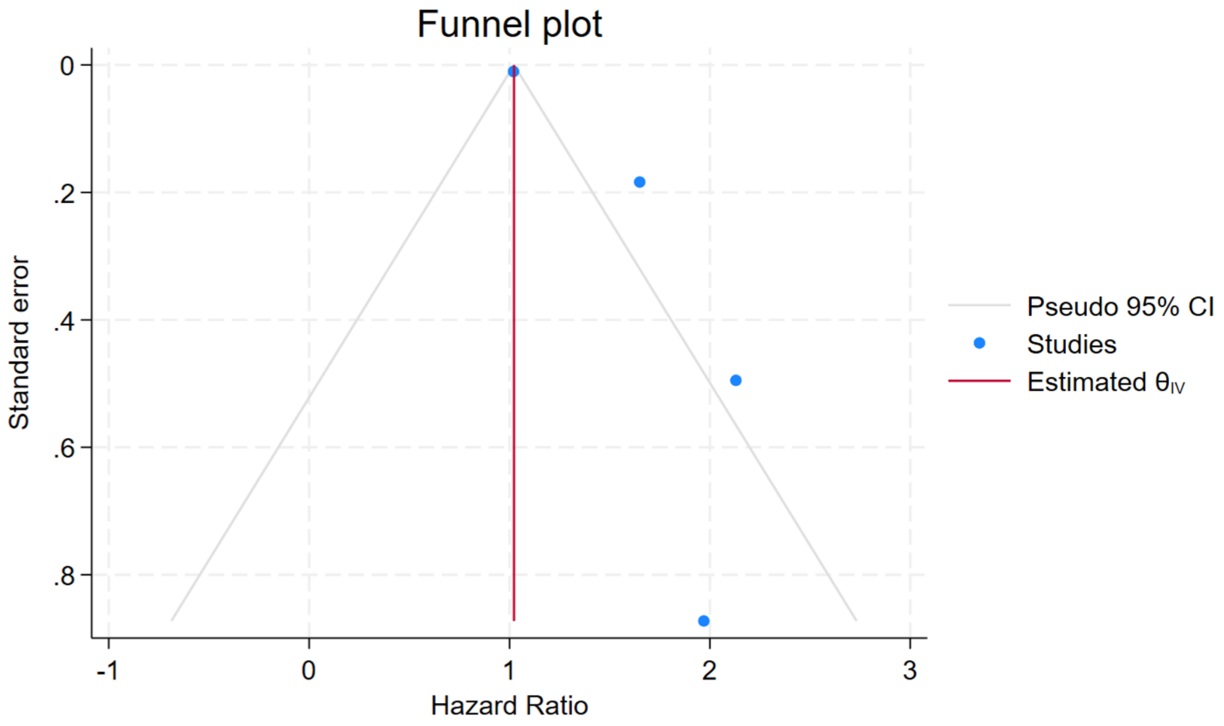

Figure S11 . Funnel plot of studies for Homocysteine Biomarker prediction of CVD among adults having Autoimmune diseases without a prior CVD history or symptom.

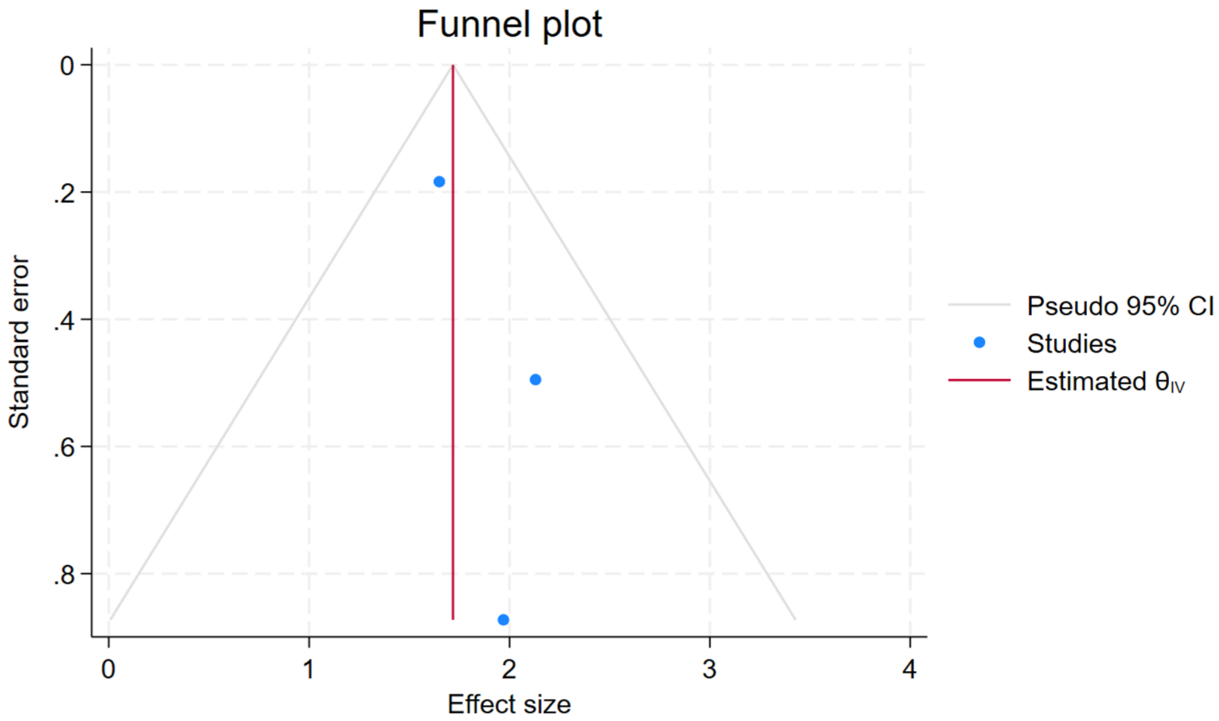

Figure S12. Funnel plot of Sensitivity Analysis of studies for Homocysteine Biomarker prediction of CVD among adults having Autoimmune diseases without a prior CVD history or symptom.

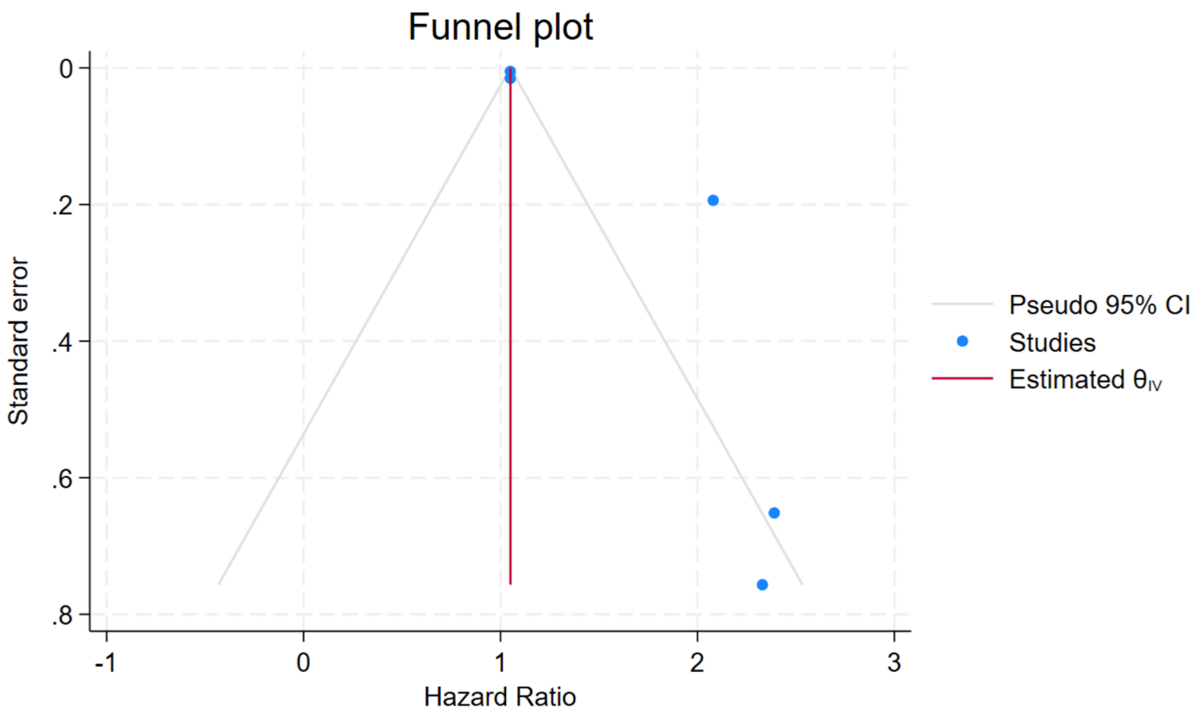

Figure S13. Funnel plot of studies for ADMA Biomarker prediction of CVD among adults having Autoimmune diseases without a prior CVD history or symptom.

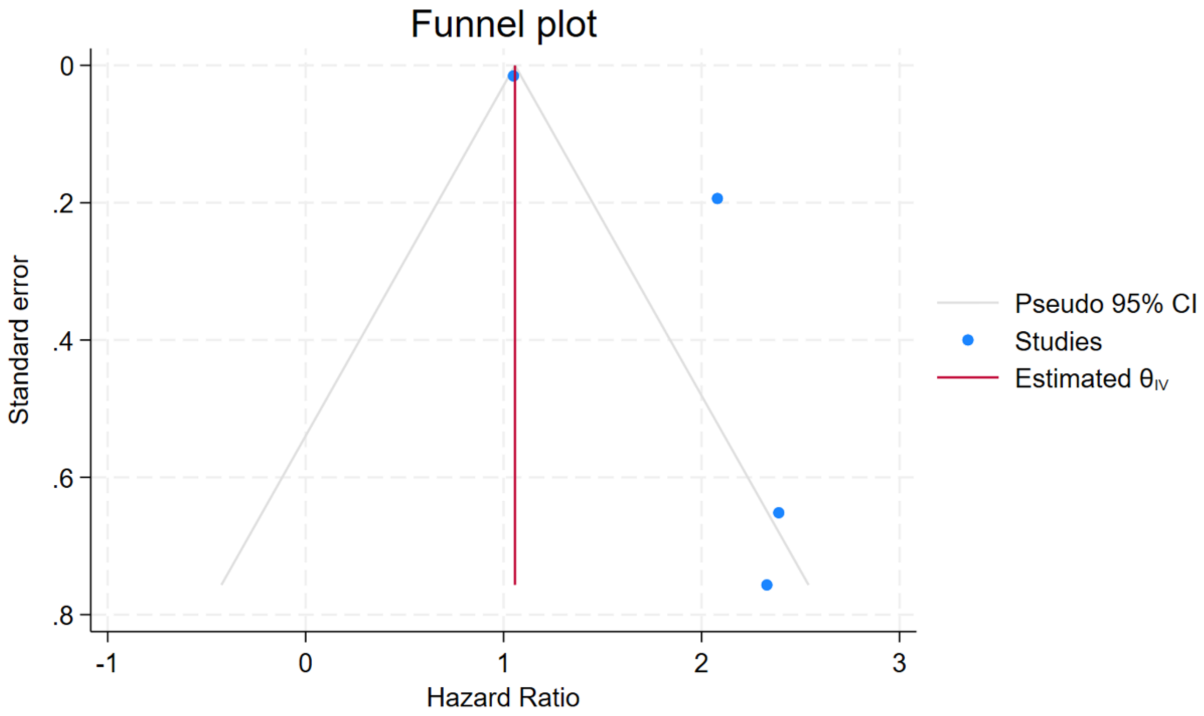

Figure S14. Funnel plot of Sensitivity Analysis of studies for ADMA Biomarker prediction of CVD among adults having Autoimmune diseases without a prior CVD history or symptom.

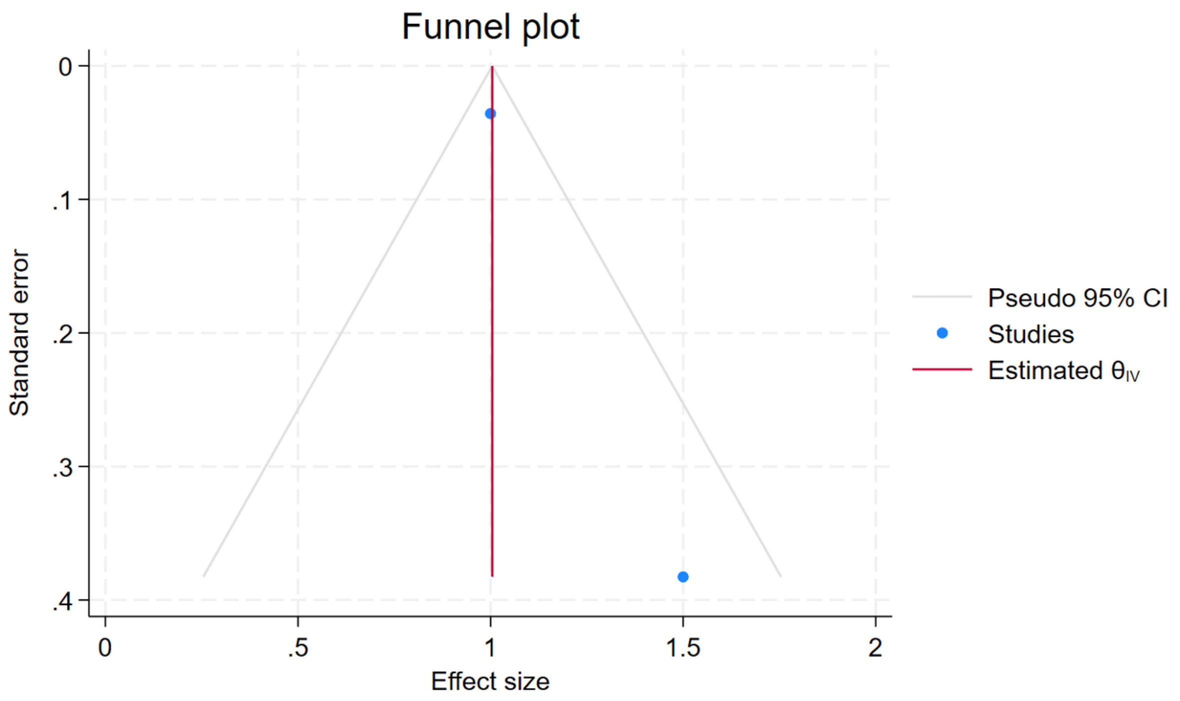

Figure S15. Funnel plot of studies for Anti-dsDNA Biomarker prediction of CVD among adults having Autoimmune diseases without a prior CVD history or symptom.

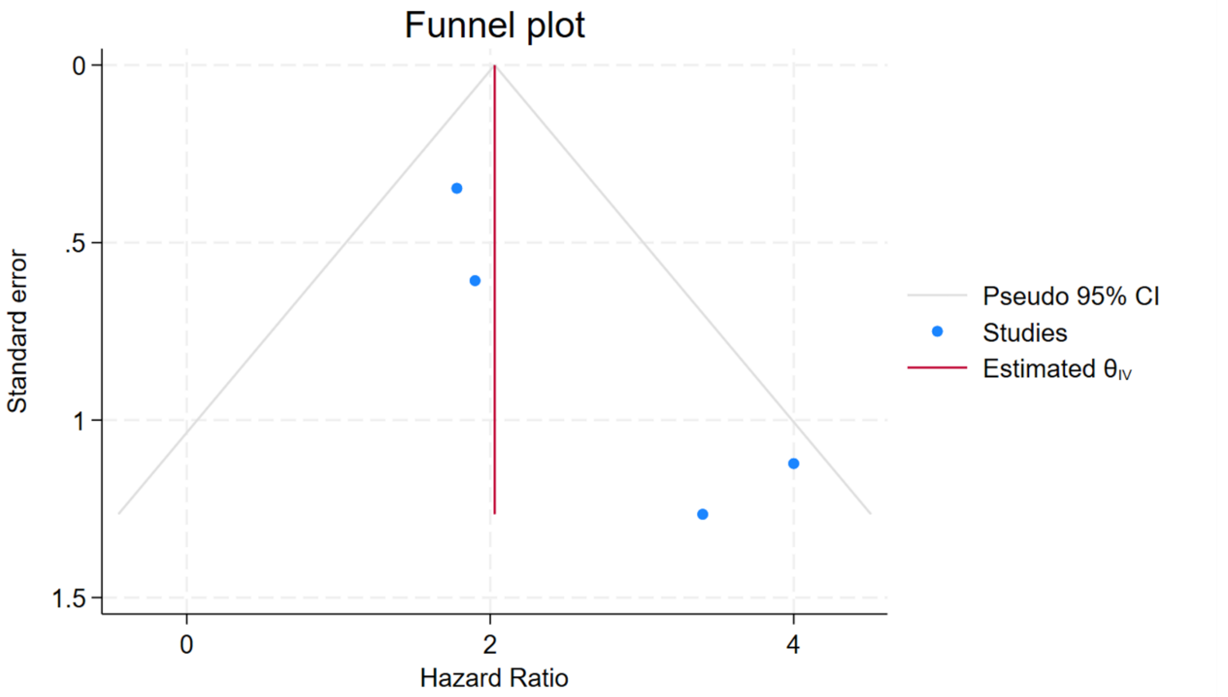

Figure S16. Funnel plot of studies for sVCAM-1 Biomarker prediction of CVD among adults having Autoimmune diseases without a prior CVD history or symptom.

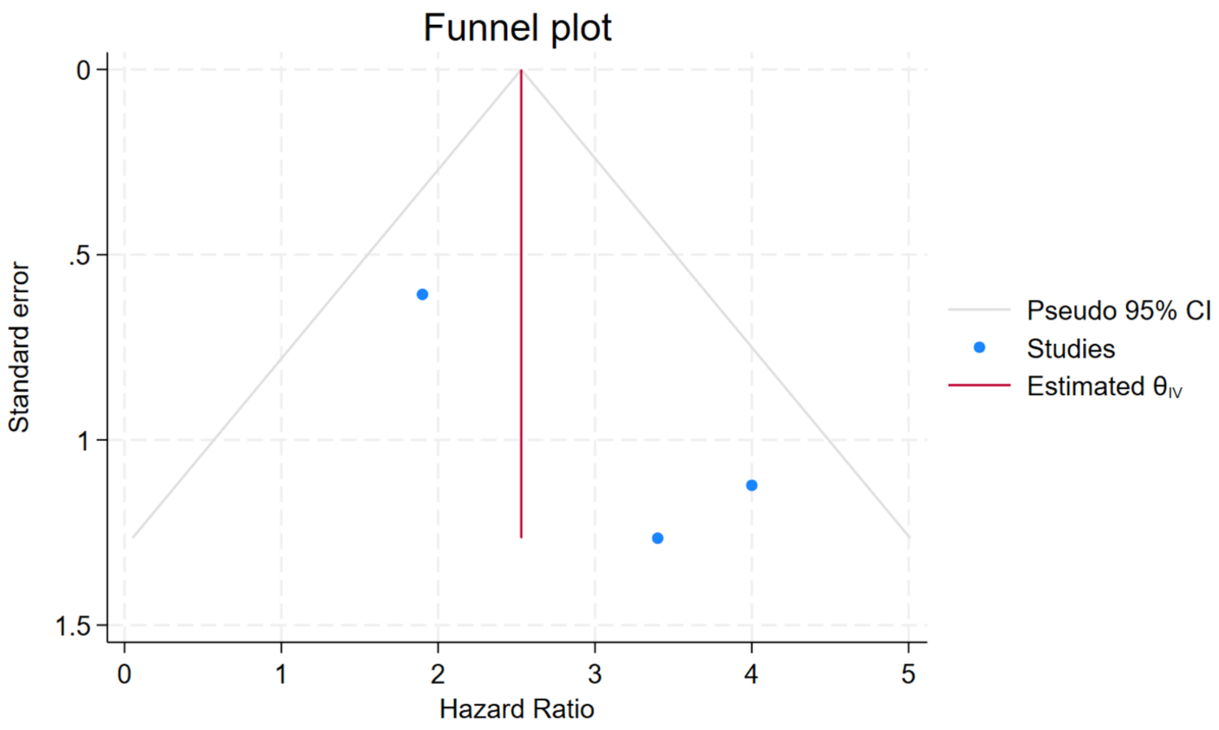

Figure S17. Funnel plot of Sensitivity Analysis studies for sVCAM-1 Biomarker prediction of CVD among adults having Autoimmune diseases without a prior CVD history or symptom.

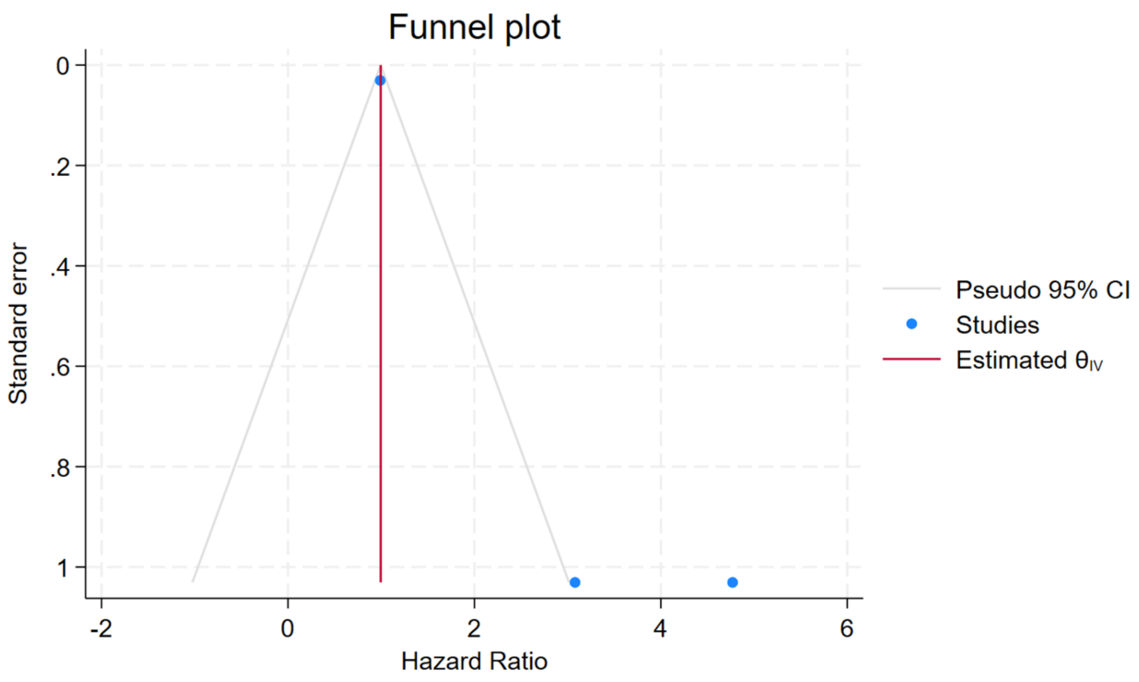

Figure S18. Funnel plot of studies for NT-ProBNP Biomarker prediction of CVD among adults having Autoimmune diseases without a prior CVD history or symptom.

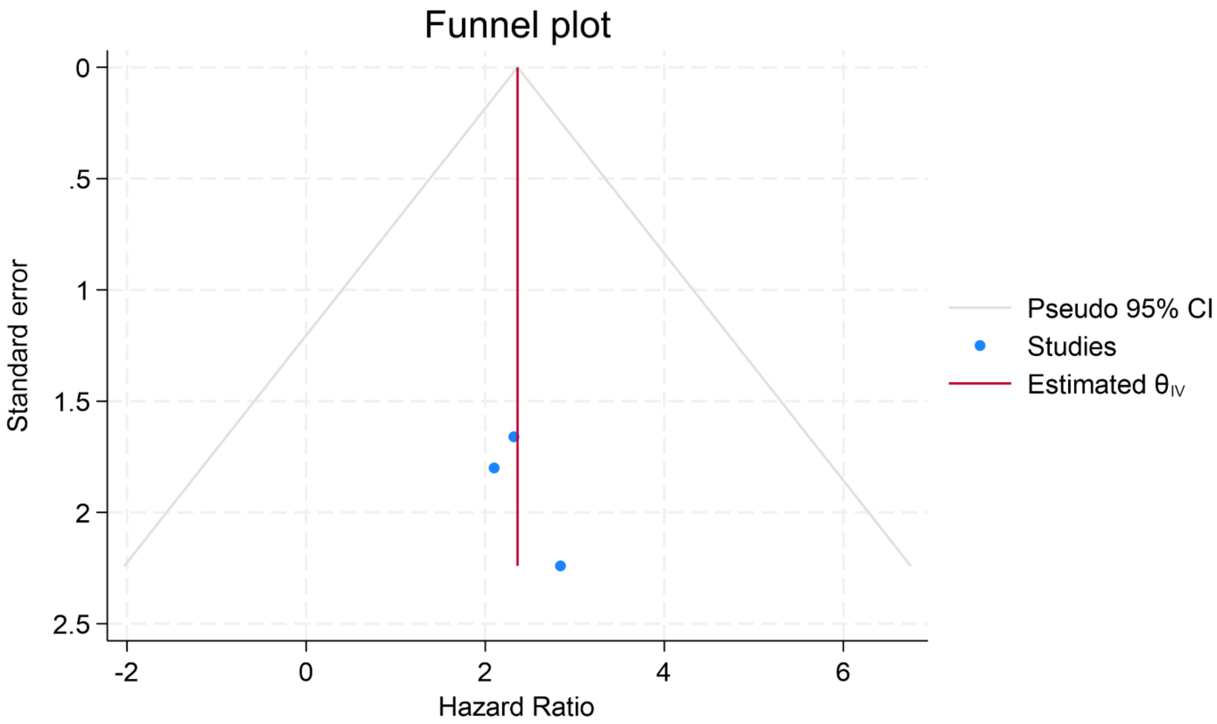

Figure S19. Funnel plot of studies for Anti-β2 glycoprotein Biomarker prediction of CVD among adults having Autoimmune diseases without a prior CVD history or symptom.

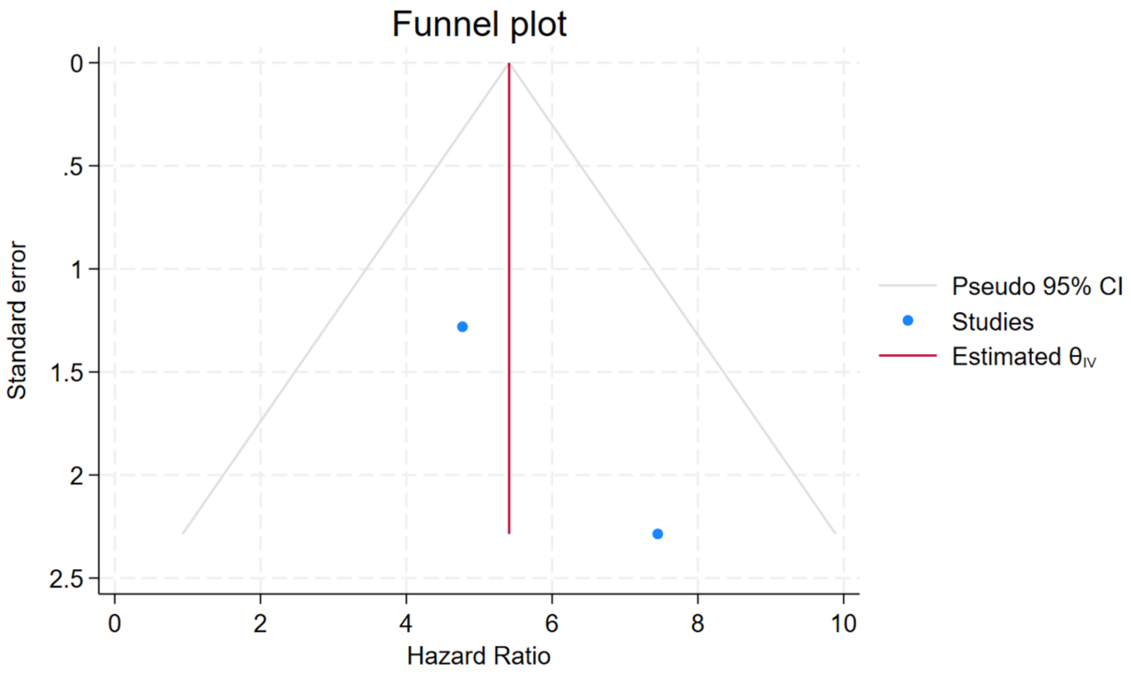

Figure S20. Funnel plot of Sensitivity Analysis of studies for Anti-β2 glycoprotein Biomarker prediction of CVD among adults having Autoimmune diseases without a prior CVD history or symptom.

2.2

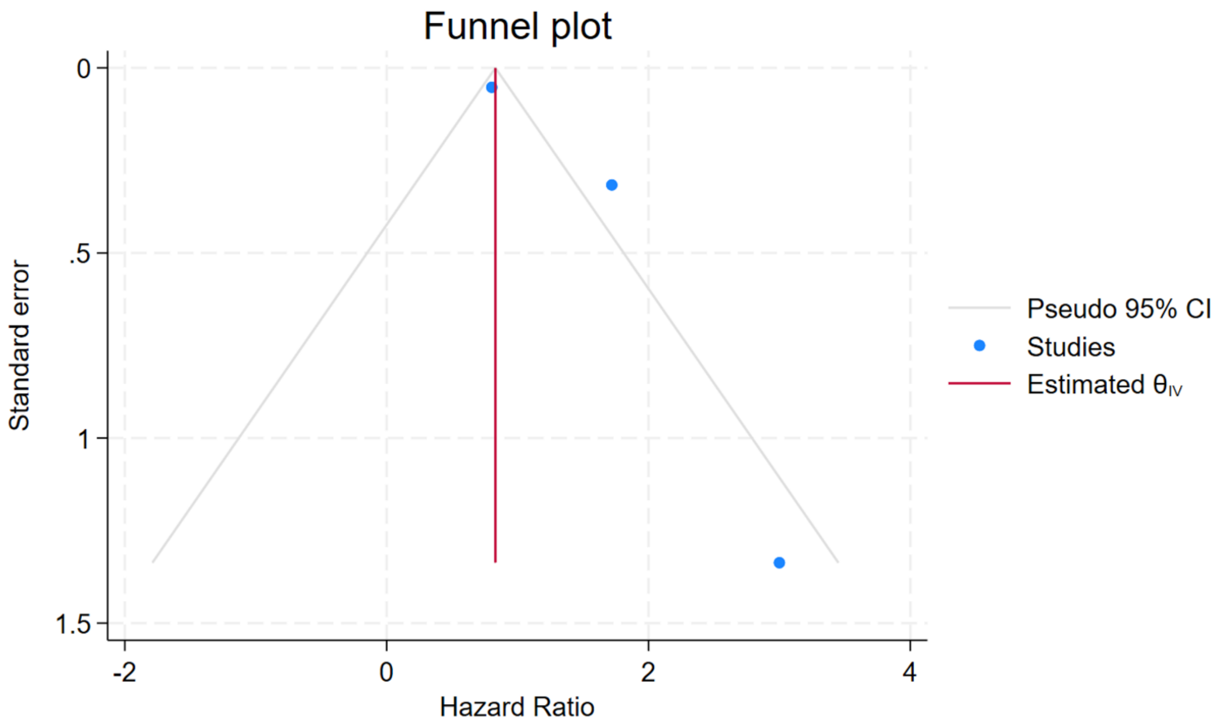

Figure S21. Funnel plot of studies for Fibrinogen Biomarker prediction of CVD among adults having Autoimmune diseases without a prior CVD history or symptom.

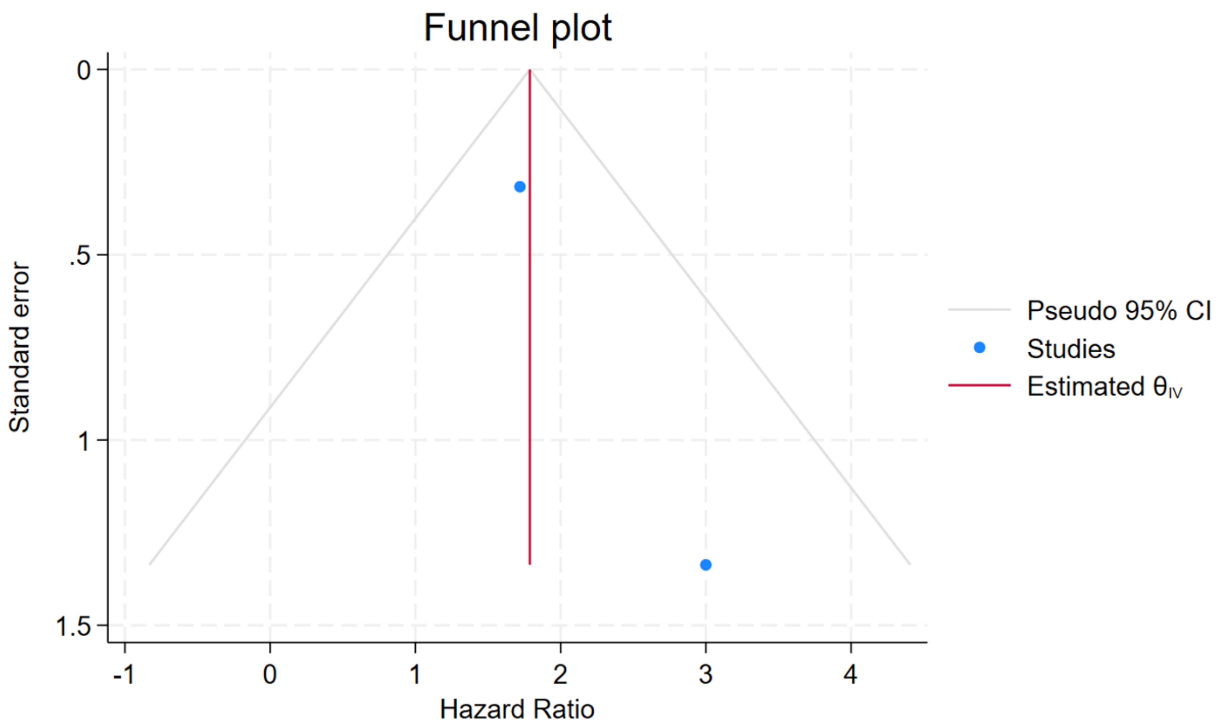

Figure S22. Funnel plot of Sensitivity Analysis of studies for Fribrinogen Biomarker prediction of CVD among adults having Autoimmune diseases without a prior CVD history or symptom.

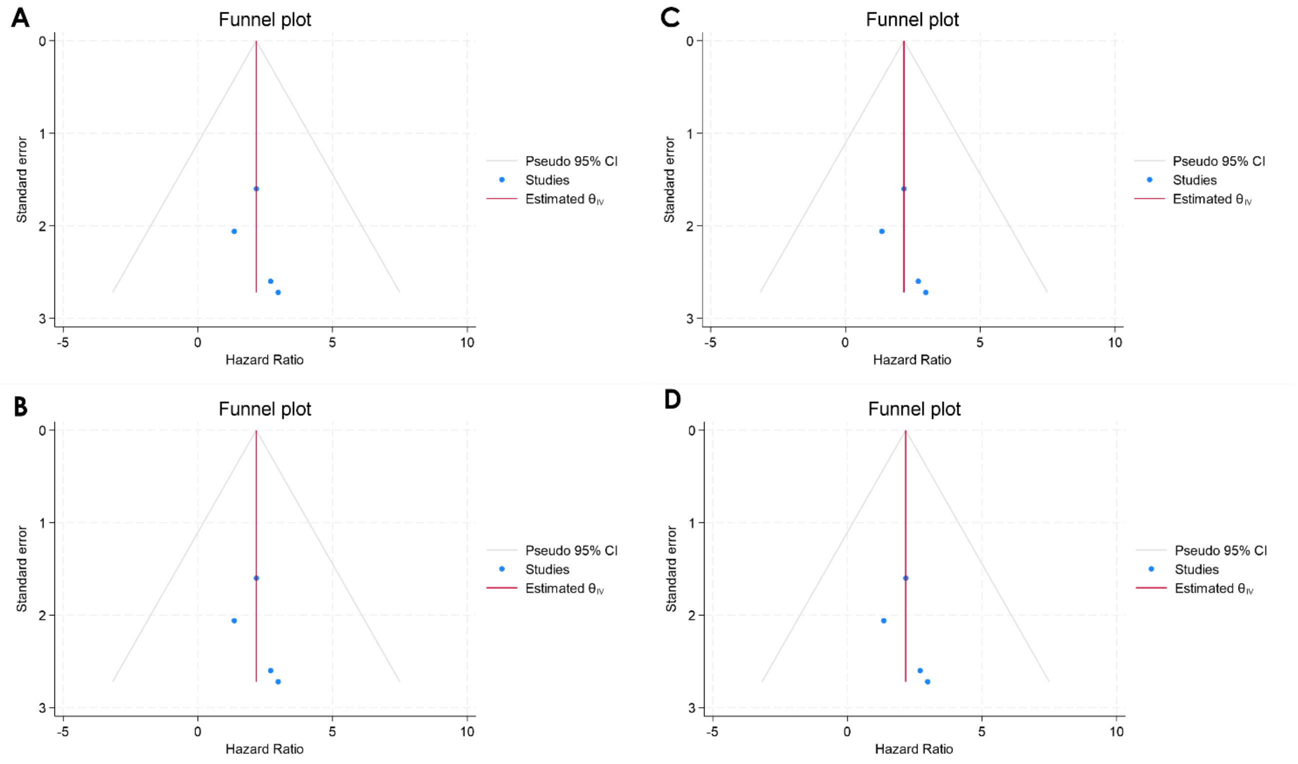

Figure S23. A. Funnel plot of studies for IgG ACL Biomarker prediction of CVD among adults having Autoimmune diseases without a prior CVD history or symptom. B IgG sensitivity Analysis Funnel Plot. C. Funnel plot of studies for IgM ACL Biomarker prediction of CVD among adults having Autoimmune diseases without a prior CVD history or symptom. D. IgM sensitivity analysis.

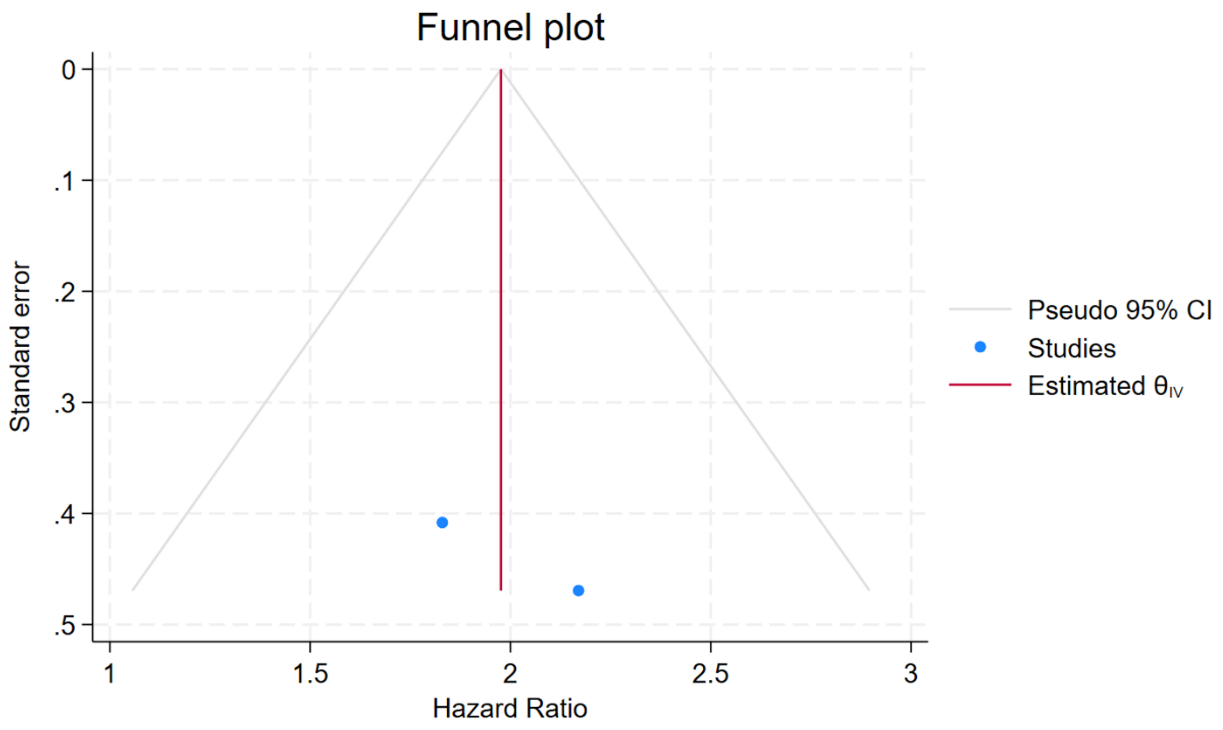

Figure S24. Funnel plot of studies for TNF-alpha Biomarker prediction of CVD among adults having Autoimmune diseases without a prior CVD history or symptom.

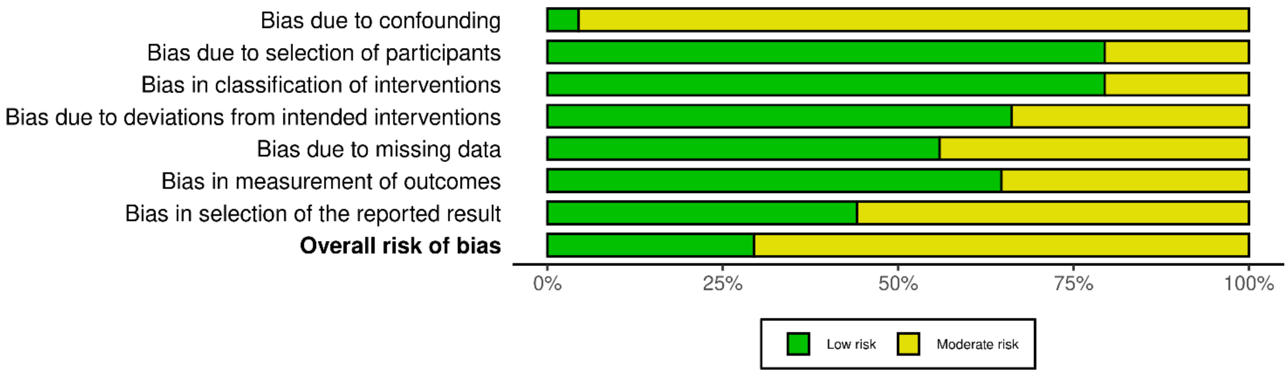

Figure S25. Risk of Bias summary

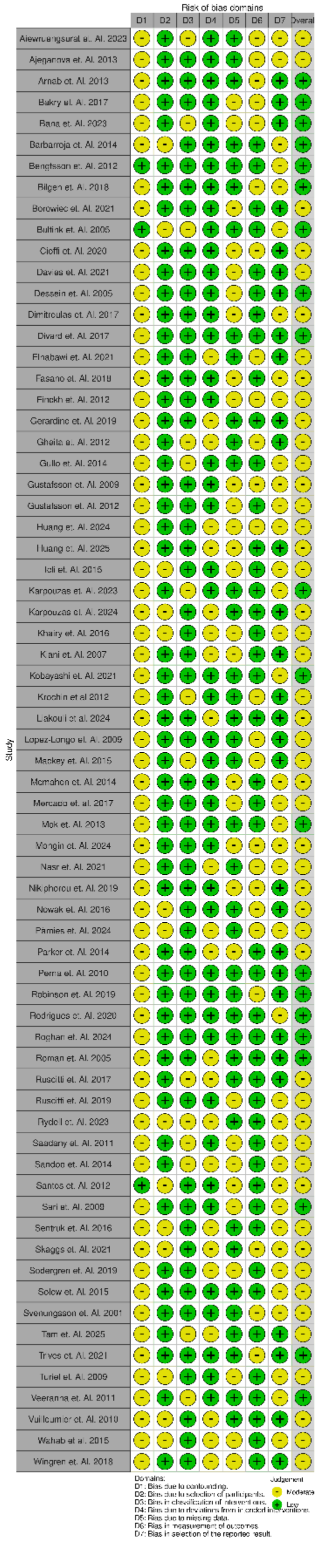

Figure S26. Risk of bias for each study.
